# Association between phenotype and deletion size in 22q11.2 microdeletion syndrome: systematic review and meta-analysis

**DOI:** 10.1186/s13023-019-1170-x

**Published:** 2019-08-09

**Authors:** M. Fernanda Rozas, Felipe Benavides, Luis León, Gabriela M. Repetto

**Affiliations:** 1 Programa de Doctorado en Ciencias e Innovación en Medicina, Facultad de Medicina, Clínica Alemana Universidad del Desarrollo, Avda Las Condes, 12461 Santiago, Chile; 2 Centro de Genética y Genómica, Facultad de Medicina, Clínica Alemana Universidad del Desarrollo, Avda Las Condes, 12438 Santiago, Chile; 3 Instituto de Ciencias Biomédicas, Facultad de Ciencias de la Salud, Universidad Autónoma de Chile, Pedro de Valdivia, 425 Santiago, Chile; 4 Present address: ThermoScientific, Santiago, Chile

**Keywords:** Congenital heart defects, Chromosome 22q11.2 deletion syndrome, DiGeorge syndrome, Meta-analysis, Palate anomalies, Systematic review, Velocardiofacial syndrome

## Abstract

**Background:**

Chromosome 22q11.2 microdeletion syndrome, a disorder caused by heterozygous loss of genetic material in chromosome region 22q11.2, has a broad range of clinical symptoms. The most common congenital anomalies involve the palate in 80% of patients, and the heart in 50–60% of them. The cause of the phenotypic variability is unknown. Patients usually harbor one of three common deletions sizes: 3, 2 and 1.5 Mb, between low copy repeats (LCR) designated A-D, A-C and A-B, respectively. This study aimed to analyze the association between these 3 deletion sizes and the presence of congenital cardiac and/or palatal malformations in individuals with this condition. A systematic review and meta-analysis were conducted, merging relevant published studies with data from Chilean patients to increase statistical power.

**Results:**

Eight articles out of 432 were included; the data from these studies was merged with our own, achieving a total of 1514 and 487 patients to evaluate cardiac and palate malformations, respectively. None of the compared deleted chromosomal segments were statistically associated with cardiac defects (OR_AB v/s AC-AD_: 0.654 [0.408–1.046]; OR _AD v/s AB-AC_: 1.291 [0.860–1.939]) or palate anomalies (OR_AB v/s AC-AD_: 1.731 [0.708–4.234]; OR _AD v/s AB-AC_: 0.628 [0.286–1.382]).

**Conclusions:**

The lack of association between deletion size and CHD or PA found in this meta-analysis suggests that deletion size does not explain the incomplete penetrance of these 2 major manifestations, and that the critical region for the development of heart and palatal abnormalities is within LCR A-B, the smallest region of overlap among the three deletion sizes.

**Electronic supplementary material:**

The online version of this article (10.1186/s13023-019-1170-x) contains supplementary material, which is available to authorized users.

## Background

Chromosome 22q11.2 deletion syndrome (22q11.2DS) (MeSH Term “DiGeorge Syndrome”; MIM #188400, #192430) is a complex disorder that includes multiple congenital and neurodevelopmental anomalies. As the name implies, it is caused by a deletion in chromosome region 22q11.2. The syndrome presents phenotypic variability, which at first led it to be misidentified as several different pathological entities such as DiGeorge, conotruncal anomaly face, and velocardiofacial syndromes [1]. The most common manifestations are palate anomalies (PA), congenital heart defects (CHD), distinctive craniofacial features, learning difficulties, cognitive deficits and psychiatric morbidity [1]. Among rare disorders, 22q11.2DS is relatively frequent, the estimated incidence is 1 in every 4000 live births and approximately 80–90% of cases are de novo [1].

**Fig. 1:**
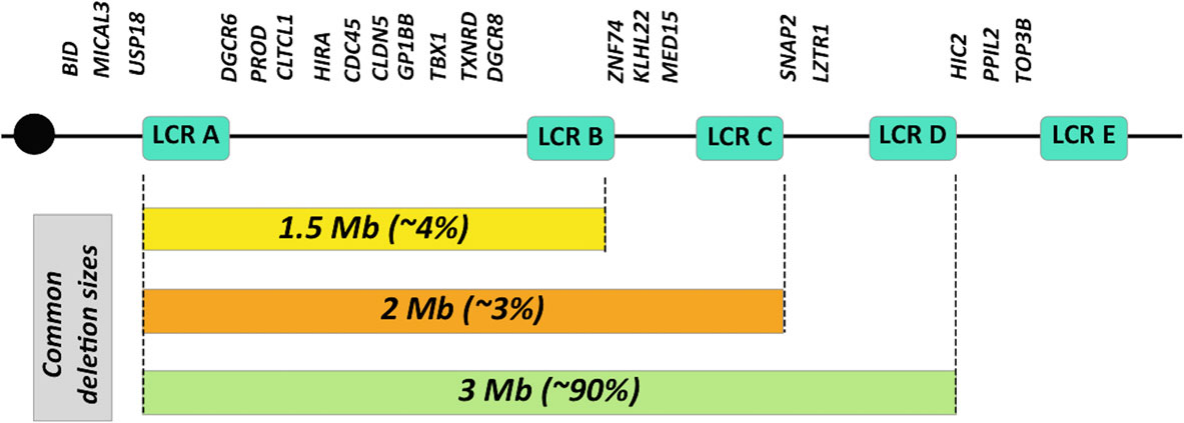
Schematic overview of the chromosome 22q11.2 region. Centromere is represented by the black circle. LCR22 A to E are illustrated by the green boxes. Horizontal bars below the map represent the most common deletions at 22q11.2 region and their frequencies

There are several low copy repeats (LCR) in the common deletion region, designated A to D, and they are susceptible to rearrangements (Fig. 1). The deletions are caused by non-allelic homologous recombination that creates deletions of variable sizes, where 3 Mb, 2 MB, and 1.5 Mb are the most common. These sizes correspond to deletions flanked by LCR-A to D, LCR-A to C and LCR-A to B, respectively [2]. Each deletion contains different genes [2, 3], but there is a minimal region of overlap, between LCRs A and B. Therefore, patients with any of the three common deletion types share haploinsufficiency for the genes between these two proximal LCRs. Among these genes is *TBX1*, which encodes for the transcription factor T-box 1 [4]. Haploinsufficiency of *TBX1* is considered the major contributor to the 22q11.2DS phenotype, as it has been associated with CHD and PA [4–6]. Other genes such as *HIRA*, *UFD1L* (located between LCR A-B) and *CRKL* (LCR C-D) have also been identified as candidate genes involved in cardiac malformations [4], but their role is less certain.

Severity of clinical manifestations may be associated with deletion size, since, in large deletions, more genes are involved. Alternatively, major causative genes could be in the minimal region of overlap, while modifier genes could be in LCR B-C or LCR C-D and contribute to the diversity in observed phenotypes. This potential association could have prognostic implications and contribute to understanding the underlying disease mechanisms. Several studies have looked at the association between deletion size and phenotype, but none report statistical analysis [7, 8]. Methodological limitations of these publications include dissimilar sample sizes, different clinical assessments and molecular studies used, differences in ascertainment sources and the fact that most individuals have the common 3 Mb deletion.

This study analyzed the association between deletion size and common phenotypic features (CHD or PA), merging data from literature review and our own results to increase statistical power and calculate a pooled risk estimator.

## Results

**Fig. 2:**
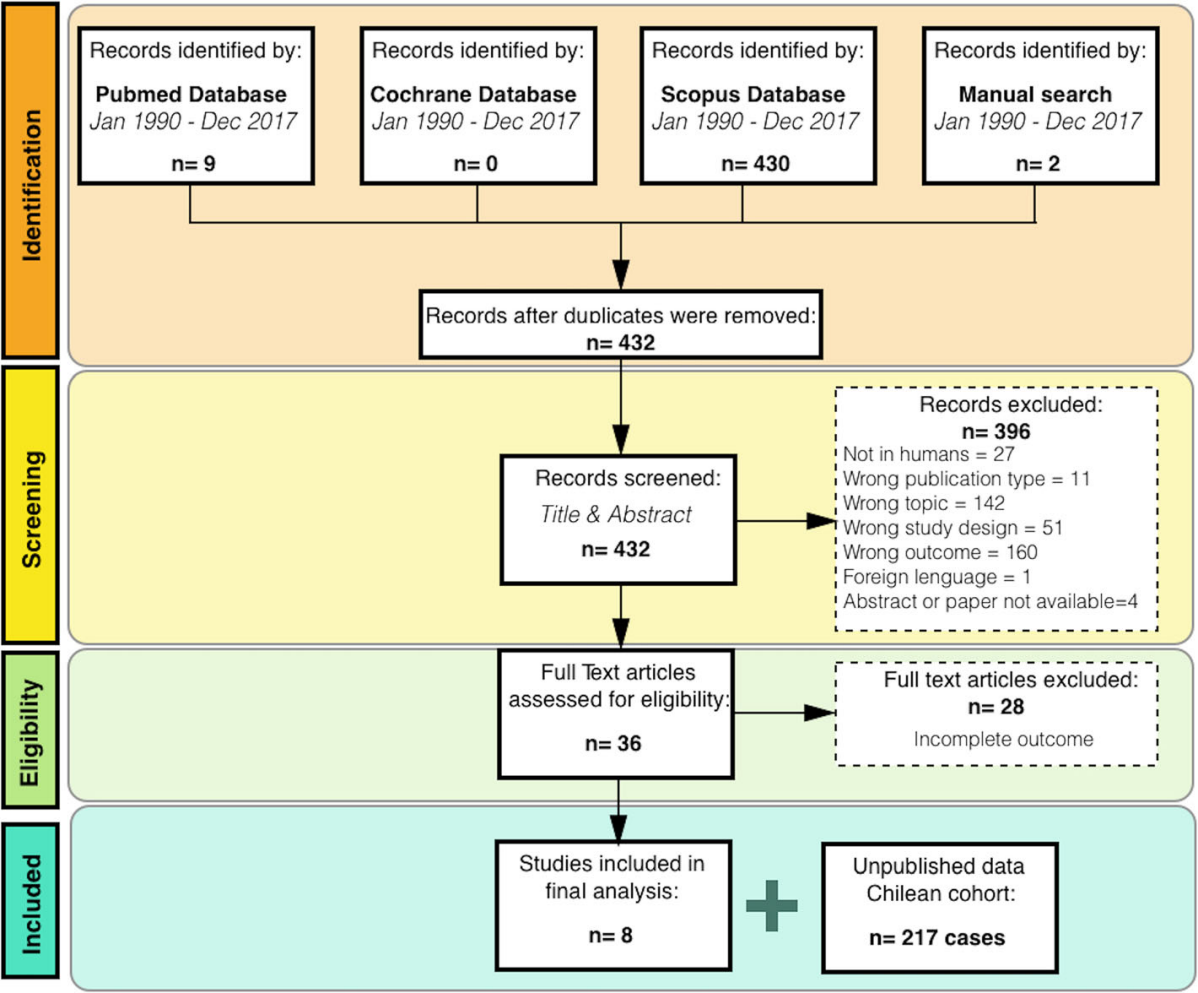
Flow diagram of study selection. Solid lines represent the path for included studies. Dashed lines represent the path for excluded studies

The systematic literature review yielded 432 unique citations (Additional file 1), two of which were identified through manual search (Fig. 2). After the initial screening, 396 articles (92%) were excluded. The full text of the remaining 36 citations was reviewed, a total of eight articles were considered eligible and were submitted to quality assessment. All of them were considered of moderate to good quality and were included in the final analysis; the main deficit was the lack of description of the statistical methods used. Although the evaluation between phenotype and deletion size was not the primary objective of these articles, the data obtained from these studies enabled the statistical estimation of this association (Additional file 2).

Carlson et al. (1997) evaluated velocardiofacial patients from a craniofacial clinic from the United States using microsatellite markers, 24 out of 26 patients had molecular confirmation of the 22q deletion; one had an unbalanced translocation including another chromosome and was excluded from the meta-analysis [9]. Kurahashi et al. (1997) evaluated 100 patients from Japan with clinical manifestations related to 22q.11.2DS, only 49 had molecular identification of the 22q deletion, and one of them was excluded due to the presence of an unbalanced translocation [10]. Fernandez et al. (2005) evaluated inherited deletions by microsatellite analysis in 15 individuals from six families from Spain [11]. Michaelovsky et al. (2012) evaluated 142 subjects with clinical manifestations of 22q.11.2DS from a neurogenetics clinic in Israel; 110 of them were diagnosed with 22q11DS using FISH and MLPA. Five of them had deletions that were different from the three common sizes and were also excluded [7]. Wu et al. (2013) evaluated 55 patients from a cleft palate clinic in China with suspected clinical diagnosis of 22q11DS, 43 had confirmation of the deletion and were included in this analysis [12]. Since all participants were selected from a cleft palate clinic, all of them had PA, which would bias the analyses for this phenotypic component. To subside this, only CHD data from this study was considered for the meta-analysis. Monteiro et al. (2013) studied 194 patients from genetics, cardiology and cranio-facial clinics in Brazil with suspected deletions. MLPA testing confirmed the deletion in 43 of them [13]. Hwang et al. (2014) included 80 individuals from a neurodevelopmental clinic in the United States and performed digital droplet PCR to assess deletion size, and compared it with quantitative PCR in a subset of 40 participants [14]. Finally, Mlynarski et al. (2015) evaluated the association between copy number variation (CNV) and CHD using Affymetrix SNP Array v6.0 in 949 patients of European descent but did not describe palatal findings [8].

A cohort of Chilean patients with 22q11.2DS were included in the analysis [15, 16]. A subset of 217 participants had information on deletion size and clinical presentation. The phenotype status was assessed through clinical evaluation, nasopharyngoscopy, and echocardiogram for PA and CHD, respectively. Types and frequencies of these anomalies are summarized in Tables 1 and 2. Deletion size analysis in 22q11.2 region was performed using SNP array v6.0 (Affymetrix) or MLPA P250 kit (MRC-Holland).

**Table 1:** Cardiac and vascular anomalies in Chilean patients

Cardiac and vascular phenotype	n	%
Normal	92	42,4%
Ventricular septal defect	32	14,7%
Tetralogy of Fallot	32	14,7%
Tetralogy Fallot with pulmonary atresia	12	5,5%
Interrupted aortic arch	11	5,1%
Atrial septal defect	10	4,6%
Truncus arteriosus	7	3,2%
Double outlet right ventricle	2	0,9%
Right sided aortic arch /vascular ring	8	3,7%
Aberrant subclavian artery	5	2,3%
CHD type not specified	5	2,3%
Cardiac phenotype unknown	1	0,5%
Total	217	100,0%

**Table 2:** Palatal anomalies in Chilean Patients

Palatal phenotype	n	%
Normal	65	30,0%
Submucous cleft palate	46	21,2%
“Infantile” velopharyngeal incompetence (VPI)^a^	43	19,8%
Velopharyngeal incompetence	22	10,1%
Cleft soft palate	16	7,4%
Cleft soft and hard palate	9	4,1%
Cleft lip	4	1,8%
Palatal anomaly, not specified	12	5,5%
Total	217	100,0%

^a^ clinical signs of VPI, but nasopharyngoscopy not performed due to age

Deletion sized and phenotypes of each study, including the Chilean cohort, are summarized in Table 3. The total number of included patients was 1514 for cardiac and 487 for palatal phenotypes. CHD was diagnosed in 61.5% of patients, and PA in 66%. The majority (91.7%) harbored the large, 3 Mb A-D deletion; A-B deletions were present in 5.3%, and A-C in 1.5%, similar to what has been described in large clinical series (reviewed in [1]).

**Table 3:** Characteristics of included studies

	CARLSON	KURAHASHI	FERNANDEZ	MICHAELOVSKY	WU	MONTEIRO	HWANG	MLYNARSKI	REPETTO
Year	1997	1997	2005	2012	2013	2013	2014	2015	2019
Journal	American J. of Human Genetics	American J. of Medical Genetics	American J. of Medical Genetics	BMC Medical Genetics	PLOS ONE	Eur J Pediatr	BMC Medical Genetics	American J. of Human Genetics	This article
Place of subjects’ selection	Center for Craniofacial Disorders, USA	Not Reported	Red de Centros de Genetica Clınica y Molecular, Spain	Behavioral Neurogenetics Center (BNC), Israel	Center for Cleft Lip and palate; China	Crânio-Face Brazil Project/Cardiopediatric Ambulatory unit of UNICAMP Clinical Hospital	UC Davis Medical Investigation of Neuro-developmental Disorders, USA	Not Reported	Genetic Departments from terciary medical centers, Chile
Evaluation of deletion size	Polymorphic STRP markers	FISH/Southern blot	Polymorphic STRP markers	MLPA	MLPA	MLPA	Droplet digital PCR	MLPA/SNP Array	MLPA/SNP Array
Cohort (n)	26	100	15	142	55	194	95	949	217
Individuals with 22q11 deletion (n)	24	49	15	110	43	45	80	949	217
LCR A-B/1.5 Mb (n) LCR A-C/2 Mb (n) LCR A-D/3 Mb (n)	10	5	8^a^	4	3	3	3	42	12
	0	0		4	0	0	0	15	5
	13	43	7	97	40	39	74	892	200
				4/ atypical 22q11 deletion (distal)		2 /extra duplications			
Excluded cases (n)/reason	1/unbalanced translocation	1/unbalanced translocation	0	1/ atypical nested deletion	0	of 22q11 region 1 / deletion size not reported	3/ atypical 22q11 deletions	0	0
Excluded cases without CHD or PA phenotype(n)	0 for PA 0 for CHD	0 for PA 0 for CHD	3 for PA 1 for CHD	0 for PA 0 for CHD	0 for PA 0 for CHD	3 for PA 3 for CHD	77 for PA 0 for CHD	949 for PA for CHD	0 for PA for CHD

*CHD* Congenital Heart Defects, *PA* palate anomalies

^a^no discrimination between A-B and A-C deletions

**Fig. 3:**
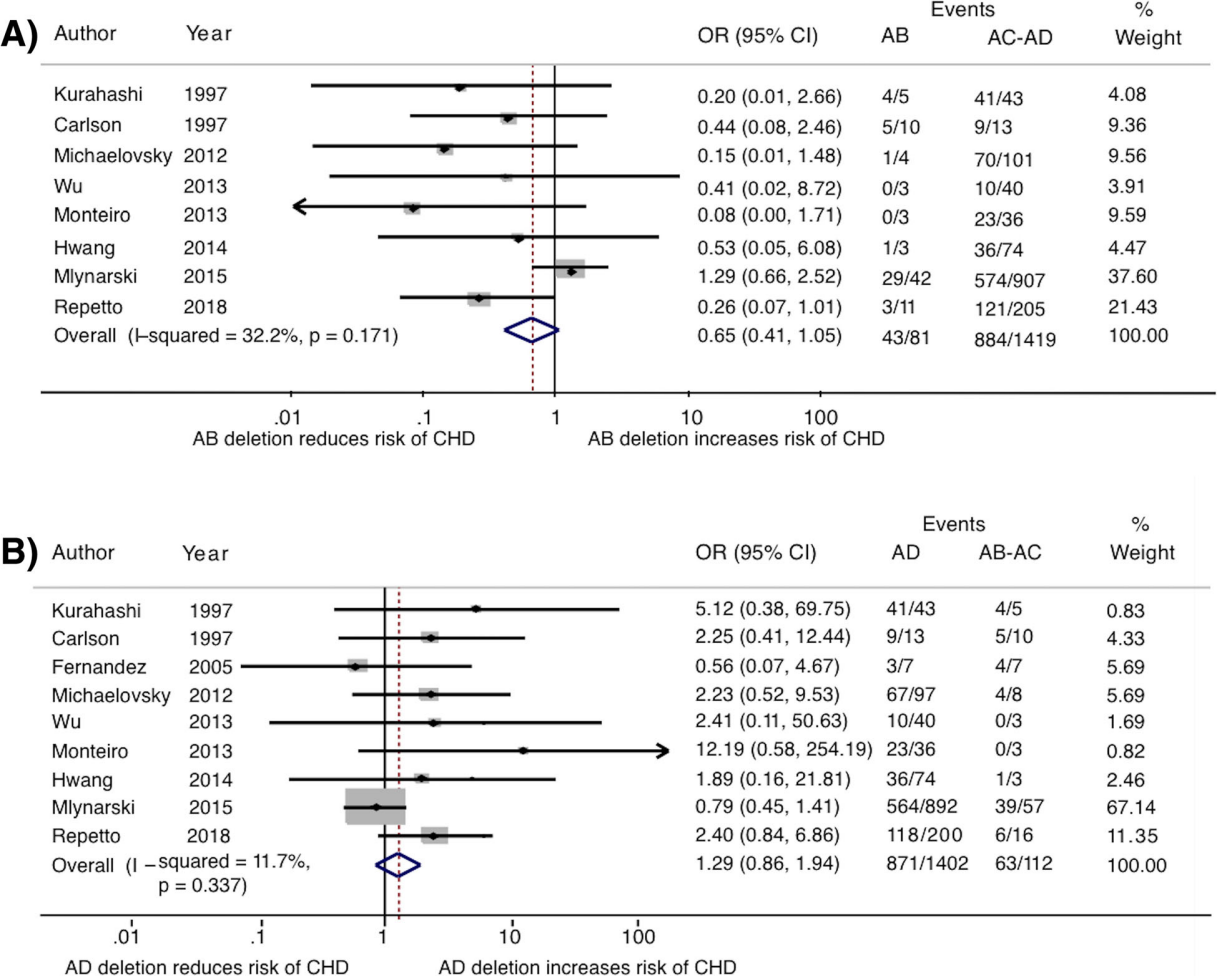
Forest plot examining the association between CHD and deletion size. OR and 95% CI of studies are shown. Pooled estimator is represented by the blue line diamond. **a** Contrast of patients with AB deletion against grouped patients with AC and AD (AC-AD) deletion. The study of Fernandez (2005) was excluded from this comparison as it did not distinguish between AB and AC deletion. **b** Contrast of patients with AD deletion against grouped patients with AB and AC (AB-AC) deletion

**Fig. 4:**
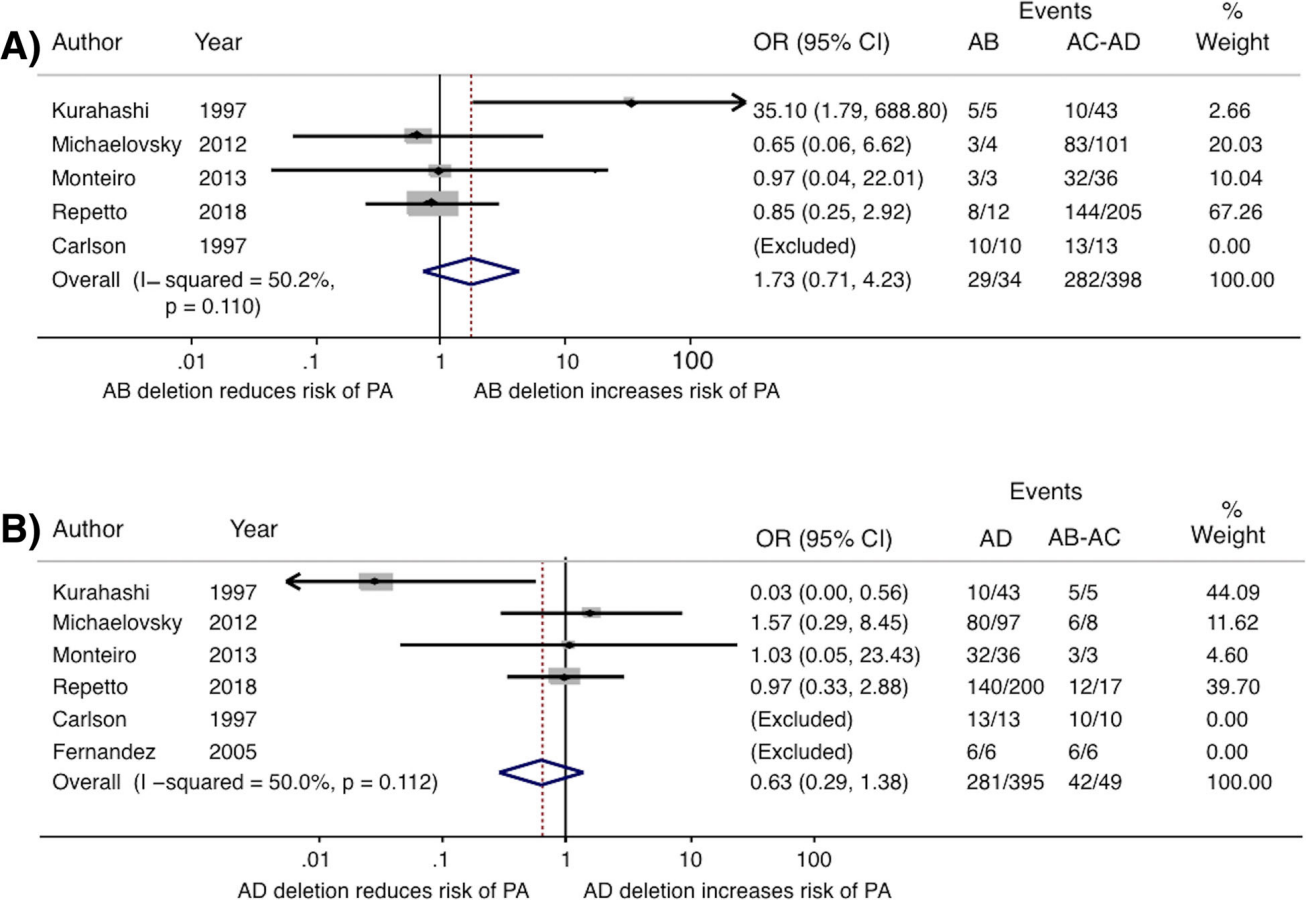
Forest plot examining the association between PA and deletion size. OR and 95% CI of studies are shown. Pooled estimator is represented by the blue line diamond. Three studies were excluded a priori from this analysis: Wu (2013) because all the patients were selected from a Center for Cleft lip and palate and all had this phenotypic manifestation; Hwang (2014) and Mlynarski (2015) as neither of these studies evaluated PA phenotype. **a** Contrast of patients with AB deletion against grouped patients with AC and AD (AC-AD) deletion. Fernandez (2005) was also excluded a priori from this analysis, as they did not differentiate between AB and AC deletions. Finally, one study was excluded during the statistical analysis, Carlson (1997), because it had all events in both arms, thus an OR of 1 that does not provide useful information to the analysis. **b** Contrast of patients with AD deletion against grouped patients with AB and AC (AB-AC) deletion. Two studies were excluded during the statistical analysis, Carlson (1997) and Fernandez (2005) because they both had all events in both arms, thus an OR of 1 that does not provide useful information to the analysis

Since included studies were not performed with identical methodology (sample selection, molecular techniques, statistical analysis), we conducted a heterogeneity test in order to measure the variability (“heterogeneity”) introduced by these differences. Low and moderate variability may exist as a result of randomness, but highly heterogeneous studies may represent underlying differences over the true effect. In such cases, statistically combined pooled OR’s should not be calculated. Statistical analyses for heterogeneity (Q and I^2^) showed that, although there was heterogeneity between the included studies, it was not enough as to hamper the calculation of a pooled estimator. I^2^ for CHD analysis was lower than 30%, indicating low interstudy variability, meanwhile for PA, I^2^ was around 50% indicating a moderate level of heterogeneity. Therefore, we proceeded with the meta- analysis for both phenotypes. The results of the individual studies, along with the overall results and their 95% confidence intervals are shown as a forest plot (Figs. 3 and 4). None of the compared LCR deletion segments were statistically associated with CHD; odds ratio (OR), 95% confidence intervals (CI) and *p*-values were: OR_AB v/s AC-AD_: 0.654 [CI: 0.408–1.046] *p* = 0.077; OR _AD v/s AB-AC_: 1.291 [CI 0.860–1.939] *p* = 0.218) or PA (CI OR_AB v/s AC-AD_: 1.731 [CI 0.708–4.234] *p* = 0.229; OR _AD v/s AB-AC_: 0.628 [CI 0.286–1.382] *p* = 0.248.

**Fig. 5:**
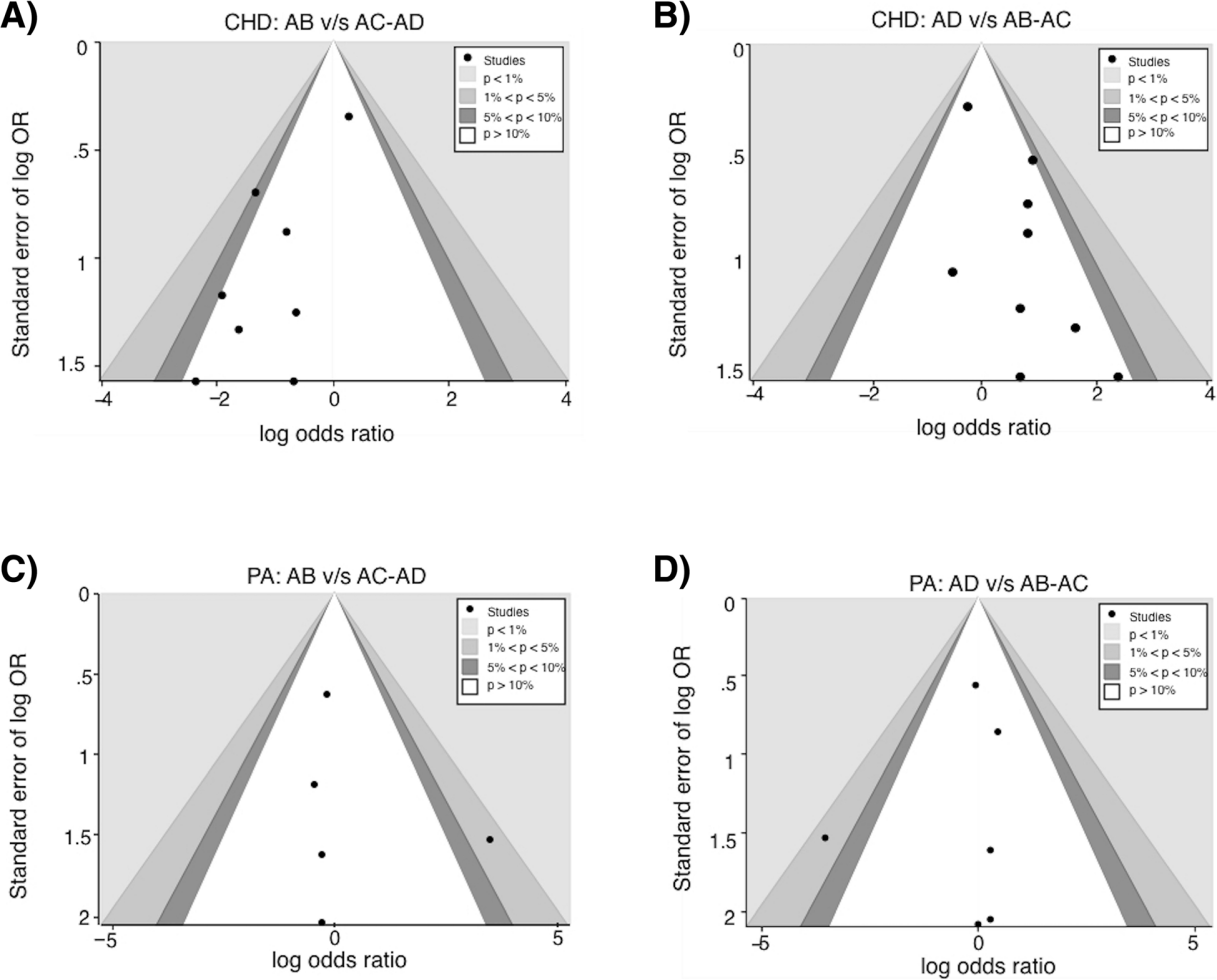
Contour-enhanced funnel plots examining publication bias. Funnel plot of effect sizes and standard errors. *p* values shown in gray shading in each panel

In all systematic reviews, publication bias is a major concern, as negative results or nonsignificant findings are less likely to be published. This type of bias was evaluated using funnel plots, where “well behaved” data (where variability comes from sampling error alone) will resemble a symmetrical inverted funnel. Publication bias analyses through funnel plots are shown in Fig. 5. All funnel plots seem to be asymmetrical, at first hand this would mean that there is publication bias, but further analysis with contour-enhanced funnel plots [17], where statistical significance of study estimates are considered (represented by the shaded regions), shows that “missing” studies, otherwise those who were allegeable not published, are in areas of statistical significance. This suggests that the asymmetry is probably due to other factors rather than publication bias.

## Discussion

This study is the first to systematically evaluate the association between the presence of major congenital anomalies and 22q11.2 deletion size. The calculated pooled ORs for all logical combinations of LCR segments showed no significant association between the presence of CHD or PA and deletion size. Several reports describe the lack of association as a fact [7, 8, 10] but to date, there was no study on which to base this claim using statistical analysis.

Sample size maybe considered as a limitation in this study, but this is true for all rare genetic diseases, such as 22q11.2DS, and illustrates the relevance of conducting meta-analysis to achieve results from larger numbers of patients. Another issue to consider is the disparity between diagnostic techniques; the studies of Carlson et al. and Kurahashi et al., were published more than 20 years ago and it could be possible that the specificity and sensitivity of the tests may not be sufficient to differentiate between the A-B and the A-C segments. This circumstance may contribute to the asymmetry observed in funnel plots. The fact that all included papers showed nonsignificant results for the association of interest supports the idea that the observed asymmetry is unlikely to represent a true publication bias, thus we did not proceed with further analyses such as trim-and-fill method.

The lack of association between deletion size and major congenital anomalies found in this study suggests that the main causative genes for CHD and PA are located in the minimal region of overlap between LCR A-B, and that genes relevant to the penetrance of CHD or PA are not located in LCR B-C or LCR C-D. Important modifying genes involving both phenotypes maybe situated outside of the 22q11.2 region.

Future studies, including other phenotypic components and technologies like massive parallel sequencing, transcriptome or epigenetics analysis may help to elucidate whether modifying phenotype genes are in fact within the 22q11.1 region.

## Conclusions

Through systematic review and meta-analysis, this study on 22q11.2 microdeletion syndrome showed no evidence of association between deletion size and the presence of congenital heart disease or palate anomalies. This suggests that the relevant genes for these manifestations are within the minimum region of overlap between the 3 common deletion sizes, and suggest the presence of modifiers elsewhere in the genome.

## Methods

### Search strategy

A systematic review was conducted using the following electronic databases: PubMed/MEDLINE, Scopus and Cochrane Library. The search was limited to papers published between 01 January 1990 to 31 December 2017, in English, French, Spanish, Portuguese, and Italian. The strategy consisted of searching the MeSH term “22q11 Deletion Syndrome” OR the entry term “DiGeorge Syndrome” in the title, abstract or keywords AND “deletion size” in all text.

### Study selection and data extraction

Two review authors (FR and GMR) independently assessed all studies for inclusion and extracted data using standardized forms. A third review author settled disagreements. The inclusion criteria for eligibility were: molecular confirmation of 22q11.2 DS through FISH, MLPA, or microarray analysis; molecular definition of deletion size through these or complementary molecular techniques; availability of clinical information, specifically the report of CHD and/or PA.

All identified citations were imported to Rayyan QCRI, a web-based tool for organizing systematic reviews [18], duplicates were excluded and the title and abstract from the identified studies were analyzed; if they appeared to be relevant, the full text was evaluated. Eligible studies were filtered by the inclusion criteria described above. Selected studies were appraised for quality using The Joanna Briggs Institute Critical Appraisal Tools for Analytical Cross-Sectional Studies [19].

Data extraction from the selected studies was performed by two independent reviewers; data was transferred to an Excel sheet which included: 1) study identification (author, journal, year), 2) molecular diagnosis tool used, 3) number of patients, 4) deletion size and location, 5) presence of CHD, 6) presence of PA, 7) other relevant observations.

### Chilean cohort

A group of Chilean patients with 22q11.2DS, ascertained from several sources (genetics, cardiology, cleft palate and neurology clinics), were included in the study; the details of this cohort have been described elsewhere [15, 16]. This study was approved by the Institutional Review Board at Facultad de Medicina Clinica Alemana Universidad del Desarrollo, Santiago, Chile.

The information from studies selected through the systematic review was merged with results from the Chilean cohort.

### Data analysis

The presence and magnitude of the associations between clinical findings and deletion size are reported as OR with their calculated 95% CI. Because A-B and A-C deletions were less frequent, data from individuals who harbored LCR A-B deletions were grouped with those with LCR A-C deletions and then compared with the larger LCR A-D deletions. As a subsequent step, LCR A-C and LCR A-D deletions were grouped and compared to the proximal LCR A-B deletion.

Total heterogeneity was measured through Cochrane’s Q, and the percentage of heterogeneity due to inter-study variation was estimated using I^2^ statistic, these values guide the methodological decision of calculating a pooled estimate if studies are of similar conditions.

Since the outcomes of interest are objective (presence/absence of phenotype versus deletion size), and represent the underlying genetic change, a single true effect can be assumed; in this scenario a single pooled OR was calculated based on Mantel-Haenszel fixed effect model. Publication bias was assessed using funnel plots. For this analysis, all cells with zero cases were replaced with a value of 0.5 in order to allow the mathematical calculation of OR, as the statistical package METAN does automatically when it encounters a cell with zero events.

Statistical analyses were performed in STATA 11 using the METAN and CONFUNNEL packages [20].

In accordance to recommendations for reporting of meta-analysis for observational studies [21], the following material is included as Additional Files: List of Citations appraised for the meta-analysis (Additional file 1); Raw data for calculation of odds ratios (Additional file 2).

## Additional files

Additional file 1: List of citations of the systematic review. Table with the information of individual studies that were identified by the systematic review, inclusion/exclusion status and justification. (PDF 455 kb)

Additional file 2: Raw data from individual studies. Table with the frequency of phenotypic outcomes cross tabulated with information on deletion size and segment of deletion. (PDF 52 kb)

## Data Availability

Study performed on already published material. The data sets from the Chilean participants analyzed during the current study are available from the corresponding author on reasonable request.

## Abbreviations

22q11.2DS: Chromosome 22q11.2 deletion syndrome
CHD: Congenital Heart Disease
LCR: Low Copy Repeats
PA: Palate Anomaly
