# Supplementary material for: Association between phenotype and deletion size in 22q11.2 microdeletion syndrome: systematic review and meta-analysis
Source: Orphanet J Rare Dis. 2019 Aug 9;14:195. doi: 10.1186/s13023-019-1170-x (PMC6688301; doi:10.1186/s13023-019-1170-x)
Supplement: Supplementary file 1 — List of citations of the systematic review. Table with the information of individual studies that were identified by the systematic review, inclusion/exclusion status and justification. (PDF 455 kb) [file 13023_2019_1170_MOESM1_ESM.pdf]

Authors	Title	Year	Source title	Vol.	Page start	Page end	*FT/ T&A	Included/ excluded	Justification
**Nugent N., McGillivary A., Earley M.J.**	22q11 chromosome abnormalities and the cleft service	2010	Journal of Plastic, Reconstructive and Aesthetic Surgery	63	598	602	T&A	Excluded	wrong outcome
**Bassett A.S., Chow E.W.C.**	22q11 deletion syndrome: A genetic subtype of schizophrenia	1999	Biological Psychiatry	46	882	891	T&A	Excluded	wrong study design
**Squarcione C., Torti M.C., Di Fabio F., Biondi M.**	22q11 deletion syndrome: A review of the neuropsychiatric features and their neurobiological basis	2013	Neuropsychiatric Disease and Treatment	9	1873	1883	T&A	Excluded	wrong study design
**Ramírez-Cheyne J., Forero-Forero J.V., González-Teshima L.Y., Madrid A., Saldarriaga W.**	22q11 deletion syndrome: embryology and diagnostic algorithm [Síndrome de deleción 22q11: bases embriológicas y algoritmo diagnóstico]	2016	Revista Colombiana de Cardiologia	23	443	452	T&A	Excluded	wrong study design
**Cisarik F., Holubová V.**	22q11 microdeletion in a group of patients selected according to the malformation spectrum of CATCH 22 syndrome [Mikrodelécia 22q11 v súbore pacientov vybraných podl'a spektra vývojových chýb syndrómu CATCH 22]	2001	Lekarsky Obzor	50	373	376	T&A	Excluded	wrong outcome
**Mercer-Rosa L., Paridon S.M., Fogel M.A., Rychik J., Tanel R.E., Zhao H., Zhang X., Yang W., Shults J., Goldmuntz E.**	22q11.2 deletion status and disease burden in children and adolescents with tetralogy of Fallot	2015	Circulation: Cardiovascular Genetics	8	74	81	T&A	Excluded	wrong outcome
**Rosa R.F.M., Zen P.R.G., Graziadio C., Paskulin G.A.**	22q11.2 deletion syndrome and congenital heart defects [Síndrome de deleção 22q11.2 e cardiopatias congênitas]	2011	Revista Paulista de Pediatria	29	251	260	T&A	Excluded	wrong study design
**Antshel K.M., Kates W.R., Roizen N., Fremont W., Shprintzen R.J.**	22q11.2 Deletion Syndrome: Genetics, neuroanatomy and cognitive/behavioral features	2005	Child Neuropsychology	11	5	19	T&A	Excluded	wrong study design

**Garavelli L., Rosato S., Wischmeijer A., Gelmini C., Esposito A., Mazzanti L., Franchi F., De Crescenzo A., Palumbo O., Carella M., Riccio A.**	22q11.2 distal deletion syndrome: Description of a new case with truncus arteriosus type 2 and review	2011	Molecular Syndromology	2	35	44	T&A	Excluded	wrong study design
**Ben-Shachar S., Ou Z., Shaw C.A., Belmont J.W., Patel M.S., Hummel M., Amato S., Tartaglia N., Berg J., Sutton V.R., Lalani S.R., Chinault A. ., Cheung S.W., Lupski J.R., Patel A.**	22q11.2 Distal Deletion: A Recurrent Genomic Disorder Distinct from DiGeorge Syndrome and Velocardiofacial Syndrome	2008	American Journal of Human Genetics	82	214	221	T&A	Excluded	wrong topic
**Kim H.J., Jo H.S., Yoo E.-G., Chung I.H., Kim S.W., Lee K.H., Chang Y.H.**	22q11.2 Microduplication with thyroid hemiagenesis	2013	Hormone Research in Paediatrics	79	243	249	T&A	Excluded	wrong topic
**Koç A., Karaer K., Ergün M.A., Yirmibeş-Karaoğuz M., Kan D., Cansu A., Perçin F.**	A case with a ring chromosome 22	2008	Turkish Journal of Pediatrics	50	193	196	T&A	Excluded	wrong topic
**Daw S.C.M., Taylor C., Kraman M., Call K., Mao J.-I., Schuffenhauer S., Meitinger T., Lipson T., Goodship J., Scambler P.**	A common region of 10p deleted in DiGeorge and velocardiofacial syndromes	1996	Nature Genetics	13	458	460	T&A	Excluded	wrong outcome
**Campbell L.E., Stevens A., Daly E., Toal F., Azuma R., Karmiloff-Smith A., Murphy D.G.M., Murphy K.C.**	A comparative study of cognition and brain anatomy between two neurodevelopmental disorders: 22q11.2 deletion syndrome and Williams syndrome	2009	Neuropsychologia	47	1034	1044	T&A	Excluded	wrong outcome
**McCarthy C.S., Ramprashad A., Thompson C., Botti J.-A., Coman I.L., Kates W.R.**	A comparison of FreeSurfer-generated data with and without manual intervention	2015	Frontiers in Neuroscience	9			T&A	Excluded	wrong topic
**Haltrich I., Pikó H., Kiss E., Tóth Z., Karcagi V., Fekete G.**	A de novo atypical ring sSMC(22) characterized by array CGH in a boy with cat-eye syndrome	2014	Molecular Cytogenetics	7			T&A	Excluded	wrong topic
**Fernández L., Nevado J., Santos F., Heine-Suñer D., Martinez-Glez V., García-Miñaur S., Palomo R., Delicado A., Pajares I.L., Palomares M., García-Guereta L., Valverde E., Hawkins F., Lapunzina P.**	A deletion and a duplication in distal 22q11.2 deletion syndrome region. Clinical implications and review	2009	BMC Medical Genetics	10			T&A	Excluded	wrong study design

**Voigt J., Papalopulu N.**	A dominant-negative form of the E3 ubiquitin ligase Cullin-1 disrupts the correct allocation of cell fate in the neural crest lineage	2006	Development	133	559	568	T&A	Excluded	no human
**Poirsier C, et al.**	A French multicenter study of over 700 patients with 22q11 deletions diagnosed using FISH or aCGH.	2016	Eur J Hum Genet		844	851	FT	Excluded	incomplete outcome
**Ellegood J., Markx S., Lerch J.P., Steadman P.E., Genç C., Provenzano F., Kushner S.A., Henkelman R.M., Karayiorgou M., Gogos J.A.**	A highly specific pattern of volumetric brain changes due to 22q11.2 deletions in both mice and humans	2014	Molecular Psychiatry	19			T&A	Excluded	wrong outcome
**Kerstjens-Frederikse W.S., Hofstra R.M.W., Van Essen A.J., Meijers J.H.C., Buys C.H.C.M.**	A Hirschsprung disease locus at 22q11?	1999	Journal of Medical Genetics	36	221	224	T&A	Excluded	wrong topic
**Gur R.E., Bassett A.S., McDonald-Mcginn D.M., Bearden C.E., Chow E., Emanuel B.S., Owen M., Swillen A., Van Den Bree M., Vermeesch J., Vorstman J.A.S., Warren S., Lehner T., Morrow B.**	A neurogenetic model for the study of schizophrenia spectrum disorders: The International 22q11.2 Deletion Syndrome Brain Behavior Consortium	2017	Molecular Psychiatry	22	1664	1672	FT	Excluded	incomplete outcome
**Simon T.J.**	A new account of the neurocognitive foundations of impairments in space, time, and number processing in children with chromosome 22q11.2 deletion syndrome	2008	Developmental Disabilities Research Reviews	14	52	58	T&A	Excluded	wrong study design
**Rauch A., Hofbeck M., Leipold G., Singer H., Pfeiffer R.A.**	A new type of 22q11.2 microdeletion associated with Digeorge syndrome	1999	Genetic Counseling	10	106	107	T&A	Excluded	wrong topic
**Ogilvie C.M., Ahn J.W., Mann K., Roberts R.G., Flinter F.**	A novel deletion in proximal 22q associated with cardiac septal defects and microcephaly: A case report	2009	Molecular Cytogenetics	2			T&A	Excluded	wrong study design
**Funke B.H., Brown A.C., Ramoni M.F., Regan M.E., Baglieri C., Finn C.T., Babcock M., Shprintzen R.J., Morrow B.E., Kucherlapati R.**	A novel, single nucleotide polymorphism-based assay to detect 22q11 deletions	2007	Genetic Testing	11	91	100	FT	Excluded	incomplete outcome

**Mikhail F.M., Descartes M., Piotrowski A., Andersson R., de Ståhl T.D., Komorowski J., Bruder C.E., Dumanski J.P., Carroll A.J.**	A previously unrecognized microdeletion syndrome on chromosome 22 Band q11.2 encompassing the BCR Gene	2007	American Journal of Medical Genetics, Part A	143	2178	2184	T&A	Excluded	wrong study design
**Schaer M., Bach Cuadra M., Tamarit L., Lazeyras F., Eliez S., Thiran J.-P.**	A Surface-based approach to quantify local cortical gyrification	2008	IEEE Transactions on Medical Imaging	27	161	170	T&A	Excluded	wrong topic
**Earls L.R., Zakharenko S.S.**	A synaptic function approach to investigating complex psychiatric diseases	2014	Neuroscientist	20	257	271	T&A	Excluded	wrong topic
**Vaz S.O., Pires R., Pires L.M., Carreira I.M., Anjos R., Maciel P., Mota-Vieira L.**	A unique phenotype in a patient with a rare triplication of the 22q11.2 region and new clinical insights of the 22q11.2 microduplication syndrome: A report of two cases	2015	BMC Pediatrics	15			T&A	Excluded	wrong topic
**Schmitt J.E., Vandekar S., Yi J., Calkins M.E., Ruparel K., Roalf D.R., Whinna D., Souders M.C., Satterwaite T.D., Prabhakaran K., Mcdonald-Mcginn D.M., Zackai E.H., Gur R.C., Emanuel B.S., Gur R.E.**	Aberrant Cortical Morphometry in the 22q11.2 Deletion Syndrome	2015	Biological Psychiatry	78	135	143	T&A	Excluded	wrong outcome
**Schaer M., Eric Schmitt J., Glaser B., Lazeyras F., Delavelle J., Eliez S.**	Abnormal patterns of cortical gyrification in velo-cardio-facial syndrome (deletion 22q11.2): An MRI study	2006	Psychiatry Research - Neuroimaging	146	1	11	T&A	Excluded	wrong outcome
**Shashi V., Muddasani S., Santos C.C., Berry M.N., Kwapil T.R., Lewandowski E., Keshavan M.S.**	Abnormalities of the corpus callosum in nonpsychotic children with chromosome 22q11 deletion syndrome	2004	NeuroImage	21	1399	1406	T&A	Excluded	wrong outcome
**Miyabara S., Ando M., Yoshida K., Saito N., Sugihara H.**	Absent aortic and pulmonary valves: Investigation of three fetal cases with cystic hygroma and review of the literature	1994	Heart and Vessels	9	49	55	T&A	Excluded	wrong topic

**Lamouroux A., Mousty E., Prodhomme O., Bigi N., Le Gac M.-P., Letouzey V., De Tayrac R., Mares P.**	Absent or hypoplastic thymus: A marker for 22q11.2 microdeletion syndrome in case of polyhydramnios [La dysgénésie thymique:marqueur de microdélétion 22q11.2 dans le bilan d'un hydramnios]	2016	Journal de Gynecologie Obstetrique et Biologie de la Reproduction	45	388	396	T&A	Excluded	wrong outcome
**Meskaldji D.E., Ottet M.-C., Cammoun L., Hagmann P., Meuli R., Eliez S., Thiran J.P., Morgenthaler S.**	Adaptive strategy for the statistical analysis of connectomes	2011	PLoS ONE	6			T&A	Excluded	wrong topic
**Nurden A.T., Nurden P.**	Advances in our understanding of the molecular basis of disorders of platelet function	2011	Journal of Thrombosis and Haemostasis	9	76	91	T&A	Excluded	wrong topic
**Jolin E.M., Weller R.A., Jessani N.R., Zackai E.H., McDonald-McGinn D.M., Weller E.B.**	Affective disorders and other psychiatric diagnoses in children and adolescents with 22q11.2 Deletion Syndrome	2009	Journal of Affective Disorders	119	177	180	T&A	Excluded	wrong outcome
**Lima K., Følling I., Eiklid K.L., Natvig S., Abrahamsen T.G.**	Age-dependent clinical problems in a Norwegian national survey of patients with the 22q11.2 deletion syndrome	2010	European Journal of Pediatrics	169	983	989	FT	Excluded	incomplete outcome
**Dennis M., Spiegler B.J., Juranek J.J., Bigler E.D., Snead O.C., Fletcher J.M.**	Age, plasticity, and homeostasis in childhood brain disorders	2013	Neuroscience and Biobehavioral Reviews	37	2760	2773	T&A	Excluded	wrong topic
**Bearden C.E., Van Erp T.G.M., Dutton R.A., Lee A.D., Simon T.J., Cannon T.D., Emanuel B.S., McDonald-Mcginn D., Zackai E.H., Thompson P.M.**	Alterations in midline cortical thickness and gyrification patterns mapped in children with 22q11.2 deletions	2009	Cerebral Cortex	19	115	126	T&A	Excluded	wrong outcome
**Váša F., Griffa A., Scariati E., Schaer M., Urben S., Eliez S., Hagmann P.**	An affected core drives network integration deficits of the structural connectome in 22q11.2 deletion syndrome	2016	NeuroImage: Clinical	10	239	249	T&A	Excluded	wrong outcome
**Millan M.J.**	An epigenetic framework for neurodevelopmental disorders: From pathogenesis to potential therapy	2013	Neuropharmacology	68	2	82	T&A	Excluded	wrong topic

**Vergés L., Vidal F., Geán E., Alemany-Schmidt A., Oliver-Bonet M., Blanco J.**	An exploratory study of predisposing genetic factors for DiGeorge/velocardiofacial syndrome	2017	Scientific Reports	7			T&A	Excluded	wrong outcome
**Shugar A.L., Shapiro J.M., Cytrynbaum C., Hedges S., Weksberg R., Fishman L.**	An increased prevalence of thyroid disease in children with 22q11.2 deletion syndrome	2015	American Journal of Medical Genetics, Part A	167	1560	1564	T&A	Excluded	wrong outcome
**Zhang J., Ma D., Wang Y., Cao L., Wu Y., Qiao F., Liu A., Li L., Lin Y., Liu G., Liu C., Hu P., Xu Z.**	Analysis of chromosome 22q11 copy number variations by multiplex ligation-dependent probe amplification for prenatal diagnosis of congenital heart defect	2015	Molecular Cytogenetics	8			T&A	Excluded	wrong outcome
**Toyoshima M., Akamatsu W., Okada Y., Ohnishi T., Balan S., Hisano Y., Iwayama Y., Toyota T., Matsumoto T., Itasaka N., Sugiyama S., Tanaka M., Yano M., Dean B., Okano H., Yoshikawa T.**	Analysis of induced pluripotent stem cells carrying 22q11.2 deletion	2016	Translational Psychiatry	6			T&A	Excluded	no human
**Crewther S.G., Kiely P.M., Kok L.-L., Crewther D.P.**	Anomalies of genetic development as predictors of oculo-visual abnormalities in velo-cardio-facial syndrome	1998	Optometry and Vision Science	75	748	757	T&A	Excluded	wrong outcome
**Fruhman G., Van den Veyver I.B.**	Applications of Array Comparative Genomic Hybridization in Obstetrics	2010	Obstetrics and Gynecology Clinics of North America	37	71	85	T&A	Excluded	wrong topic
**Bar-Shira A., Rosner G., Rosner S., Goldstein M., Orr-Urtreger A.**	Array-based comparative genome hybridization in clinical genetics	2006	Pediatric Research	60	353	358	T&A	Excluded	wrong topic
**Di Gregorio E., Gai G., Botta G., Calcia A., Pappi P., Talarico F., Savin E., Ribotta M., Zonta A., Mancini C., Giorgio E., Cavalieri S., Restagno G., Ferrero G.B., Viora E., Pasini B., Grosso E., Brusco A., Brussino A.**	Array-Comparative Genomic Hybridization Analysis in Fetuses with Major Congenital Malformations Reveals that 24% of Cases Have Pathogenic Deletions/Duplications	2015	Cytogenetic and Genome Research	147	10	16	T&A	Excluded	wrong topic

**Higashiyama R., Ohnuma T., Takebayashi Y., Hanzawa R., Shibata N., Yamamori H., Yasuda Y., Kushima I., Aleksic B., Kondo K., Ikeda M., Hashimoto R., Iwata N., Ozaki N., Arai H.**	Association of copy number polymorphisms at the promoter and translated region of COMT with Japanese patients with schizophrenia	2016	American Journal of Medical Genetics, Part B: Neuropsychiatric Genetics	171	447	457	T&A	Excluded	wrong outcome
**Chow E.W.C., Ho A., Wei C., Voormolen E.H.J., Crawley A.P., Bassett A.S.**	Association of schizophrenia in 22q11.2 deletion syndrome and gray matter volumetric deficits in the superior temporal gyrus	2011	American Journal of Psychiatry	168	522	529	T&A	Excluded	wrong outcome
**Kessler-Icekson G., Birk E., Weintraub A.Y., Barhum Y., Kotlyar V., Schlesinger H., Rockah R., Vidne B.A., Frisch A.**	Association of tetralogy of fallot with a distinct region of del22q11.2	2002	American Journal of Medical Genetics	107	294	298	FT	Excluded	incomplete outcome
**Gupta M., Bhatnagar P., Grover S., Kaur H., Baghel R., Bhasin Y., Chauhan C., Verma B., Manduva V., Mukherjee O., Purushottam M., Sharma A., Jain S., Brahmachari S.K., Kukreti R.**	Association studies of catechol-O-methyltransferase (COMT) gene with schizophrenia and response to antipsychotic treatment	2009	Pharmacogenomics	10	385	397	T&A	Excluded	wrong outcome
**Thompson C.A., Karelis J., Middleton F.A., Gentile K., Coman I.L., Radoeva P.D., Mehta R., Fremont W.P., Antshel K.M., Faraone S.V., Kates W.R.**	Associations between neurodevelopmental genes, neuroanatomy, and ultra high risk symptoms of psychosis in 22q11.2 deletion syndrome	2017	American Journal of Medical Genetics, Part B: Neuropsychiatric Genetics	174	295	314	T&A	Excluded	wrong outcome
**Antshel K.M., Peebles J., AbdulSabur N., Higgins A.M., Roizen N., Shprintzen R., Fremont W.P., Nastasi R., Kates W.R.**	Associations between performance on the Rey-Osterrieth complex figure and regional brain volumes in children with and without velocardiofacial syndrome	2008	Developmental Neuropsychology	33	601	622	T&A	Excluded	wrong outcome
**Cornish K., Wilding J.**	Attention, Genes, and Developmental Disorders	2010	Attention, Genes, and Developmental Disorders		1	576	T&A	Excluded	wrong publication type
**Nogueira S.I., Hacker A.M., Bellucco F.T.S., Christofolini D.M., Kulikowski L.D., Cernach M.C.S.P., Emanuel B.S., Melaragno M.I.**	Atypical 22q11.2 deletion in a patient with DGS/VCFS spectrum	2008	European Journal of Medical Genetics	51	226	230	T&A	Excluded	wrong topic

**Molck M.C., Vieira T.P., Sgardioli I.C., Simioni M., dos Santos A.P., Souza J., Monteiro F.P., Gil-da-Silva-Lopes V.L.**	Atypical copy number abnormalities in 22q11.2 region: Report of three cases	2013	European Journal of Medical Genetics	56	515	520	T&A	Excluded	wrong topic
**Simon T.J., Wu Z., Avants B., Zhang H., Gee J.C., Stebbins G.T.**	Atypical cortical connectivity and visuospatial cognitive impairments are related in children with chromosome 22q11.2 deletion syndrome	2008	Behavioral and Brain Functions	4			T&A	Excluded	wrong outcome
**Beaujard M.-P., Chantot S., Dubois M., Keren B., Carpentier W., Mabboux P., Whalen S., Vodovar M., Siffroi J.-P., Portnoï M.-F.**	Atypical deletion of 22q11.2: Detection using the FISH TBX1 probe and molecular characterization with high-density SNP arrays	2009	European Journal of Medical Genetics	52	321	327	T&A	Excluded	wrong topic
**Amati F., Conti E., Novelli A., Bengala M., Digilio M.C., Marino B., Giannotti A., Gabrielli O., Novelli G., Dallapiccola B.**	Atypical deletions suggest five 22q11.2 critical regions related to the DiGeorge/velo-cardio-facial syndrome	1999	European Journal of Human Genetics	7	903	909	T&A	Excluded	wrong topic
**Weiss L.A.**	Autism genetics: Emerging data from genome-wide copynumber and single nucleotide polymorphism scans	2009	Expert Review of Molecular Diagnostics	9	795	803	T&A	Excluded	wrong topic
**Reynaud R., Derain-Court J., Braunstein D., Veyrat M., Gaudart J., Giuliano F., Philip N.**	Auxological evaluation in patients with a 22q11.2 microdeletion syndrome: Normal prevalence of obesity and neonatal length and gender influence on body mass index evolution	2011	Hormone Research in Paediatrics	76	172	177	T&A	Excluded	wrong outcome
**Antshel K.M., Conchelos J., Lanzetta G., Fremont W., Kates W.R.**	Behavior and corpus callosum morphology relationships in velocardiofacial syndrome (22q11.2 deletion syndrome)	2005	Psychiatry Research - Neuroimaging	138	235	245	T&A	Excluded	wrong outcome
**Tang K.L., Antshel K.M., Fremont W.P., Kates W.R.**	Behavioral and psychiatric phenotypes in 22q11.2 deletion syndrome	2015	Journal of Developmental and Behavioral Pediatrics	36	639	650	T&A	Excluded	wrong study design
**Bruining H., Eijkemans M.J., Kas M.J., Curran S.R., Vorstman J.A., Bolton P.F.**	Behavioral signatures related to genetic disorders in autism	2014	Molecular Autism	5			T&A	Excluded	wrong topic

**Pierdominici M., Mazzetta F., Caprini E., Marziali M., Digilio M.C., Marino B., Aiuti A., Amati F., Russo G., Novelli G., Pandolfi F., Luzi G., Giovannetti A.**	Biased T-cell receptor repertoires in patients with chromosome 22q11.2 deletion syndrome (DiGeorge syndrome/velocardiofacial syndrome)	2003	Clinical and Experimental Immunology	132	323	331	T&A	Excluded	wrong outcome
**Beliaev A.M., Mundy I.**	Bilateral simultaneous testicular torsion presenting as a diagnostic dilemma	2013	BMJ Case Reports				T&A	Excluded	wrong topic
**Winger E.E., Reed J.L., Ashoush S., El-Toukhy T., Ahuja S., Taranissi M.**	Birth defect rates in women using Adalimumab (Humira(®)) to treat immunologic-based infertility in IVF patients.	2011	American journal of reproductive immunology (New York, N.Y. : 1989)	66	237	241	T&A	Excluded	wrong topic
**Van Amelsvoort T., Daly E., Henry J., Robertson D., Ng V., Owen M., Murphy K.C., Murphy D.G.M.**	Brain anatomy in adults with velocardiofacial syndrome with and without schizophrenia: Preliminary results of a structural magnetic resonance imaging study	2004	Archives of General Psychiatry	61	1085	1096	T&A	Excluded	wrong outcome
**Eisenberg D.P., Jabbi M., Berman K.F.**	Bridging the gene-behavior divide through neuroimaging deletion syndromes: Velocardiofacial (22q11.2 Deletion) and Williams (7q11.23 Deletion) syndromes	2010	NeuroImage	53	857	869	T&A	Excluded	wrong outcome
**Couser N.L., Pande C.K., Walsh J.M., Tepperberg J., Aylsworth A.S.**	Camptodactyly and the 22q11.2 deletion syndrome	2017	American Journal of Medical Genetics, Part A	173	515	518	T&A	Excluded	wrong study design
**Freyer L., Morrow B.E.**	Canonical Wnt signaling modulates Tbx1, Eya1, and Six1 expression, restricting neurogenesis in the otic vesicle	2010	Developmental Dynamics	239	1708	1722	T&A	Excluded	no human
**Jalbrzikowski M., Hamzah Ahmed K., Patel A., Jonas R., Kushan L., Chow C., Bearden C.E.**	Categorical Versus Dimensional Approaches to Autism-Associated Intermediate Phenotypes in 22q11.2 Microdeletion Syndrome	2017	Biological Psychiatry: Cognitive Neuroscience and Neuroimaging	2	53	65	T&A	Excluded	wrong outcome

**Oppenheimer A.G., Fulmer S., Shifteh K., Chang J.-K., Brook A., Shanske A.L., Shprintzen R.J.**	Cervical vascular and upper airway asymmetry in velo-cardio-facial syndrome: Correlation of nasopharyngoscopy with MRA	2010	International Journal of Pediatric Otorhinolaryngolog y	74	619	625	T&A	Excluded	wrong outcome
**Wu D., Chen Y., Xu C., Wang K., Wang H., Zheng F., Ma D., Wang G.**	Characteristic Face: A Key Indicator for Direct Diagnosis of 22q11.2 Deletions in Chinese Velocardiofacial Syndrome Patients	2013	PLoS ONE	8			FT	Included	
**Volpe P., Paladini D., Marasini M., Buonadonna A.L., Russo M.G., Caruso G., Marzullo A., Arciprete P., Martinelli P., Gentile M.**	Characteristics, associations and outcome of absent pulmonary valve syndrome in the fetus	2004	Ultrasound in Obstetrics and Gynecology	24	623	628	T&A	Excluded	wrong topic
**Landis B.J., Cooper D.S., Hinton R.B.**	CHD associated with syndromic diagnoses: Peri-operative risk factors and early outcomes	2014	Cardiology in the Young	26	30	52	T&A	Excluded	wrong topic
**Magoulas P.L., El-Hattab A.W.**	Chromosome 15q24 microdeletion syndrome	2012	Orphanet Journal of Rare Diseases	7			T&A	Excluded	wrong topic
**Jarzembowski J.A.**	Chromosome 22q AbnormaLities	2014	Pathobiology of Human Disease: A Dynamic Encyclopedia of Disease Mechanisms		184	184	T&A	Excluded	wrong publication type
**Eliez S., Blasey C.M.**	Chromosome 22q11 deletion and brain structure [5]	2001	British Journal of Psychiatry	179	270		T&A	Excluded	wrong publication type
**Halder A., Jain M., Chaudhary I., Varma B.**	Chromosome 22q11.2 microdeletion in monozygotic twins with discordant phenotype and deletion size	2012	Molecular Cytogenetics	5			T&A	Excluded	wrong study design
**Molesky M.G.**	Chromosome 22q11.2 microdeletion syndrome	2011	Neonatal Network	30	304	311	T&A	Excluded	wrong study design

**Morrow B.E.**	Chromosome 22q11.2 rearrangement disorders	2006	Genomic Disorders: The Genomic Basis of Disease		193	206	T&A	Excluded	wrong publication type
**Dufour F., Schaer M., Debbané M., Farhoumand R., Glaser B., Eliez S.**	Cingulate gyral reductions are related to low executive functioning and psychotic symptoms in 22q11.2 deletion syndrome	2008	Neuropsychologia	46	2986	2992	T&A	Excluded	wrong outcome
**Huang L., Xie Y., Zhou Y., Luo Y., Huang X., Xu Z., Cai D., Fang Q.**	Clinical and molecular cytogenetic studies of an unrecognised 22q11.2 deletion in three families	2015	Experimental and Therapeutic Medicine	9	823	828	T&A	Excluded	wrong study design
**Christopoulou G., Sismani C., Sakellariou M., Saklamaki M., Athanassiou V., Velissariou V.**	Clinical and molecular description of the prenatal diagnosis of a fetus with a maternally inherited microduplication 22q11.2 of 2.5Mb	2013	Gene	527	694	697	T&A	Excluded	wrong topic
**Costain G., Bassett A.S.**	Clinical applications of schizophrenia genetics: Genetic diagnosis, risk, and counseling in the molecular era	2012	Application of Clinical Genetics	5	1	18	T&A	Excluded	wrong study design
**Azancot A., Eydoux P., Vuillard E., Cusin V., Baumann C., Blot P.**	Clinical spectrum of antenatal tetralogy of fallot [Spectre clinique de la tetralogie de fallot antenatale]	2000	Archives des Maladies du Coeur et des Vaisseaux	93	587	593	T&A	Excluded	wrong topic
**Roberts C., Daw S.C.M., Halford S., Scambler P.J.**	Cloning and developmental expression analysis of chick Hira (Chira), a candidate gene for DiGeorge syndrome	1997	Human Molecular Genetics	6	237	245	T&A	Excluded	no human
**Li D., Tekin M., Buch M., Fan Y.-S.**	Co-existence of other copy number variations with 22q11.2 deletion or duplication: A modifier for variable phenotypes of the syndrome?	2012	Molecular Cytogenetics	5			T&A	Excluded	wrong study design

**Meechan D.W., Rutz H.L.H., Fralish M.S., Maynard T.M., Rothblat L.A., Lamantia A.-S.**	Cognitive ability is associated with altered medial frontal cortical circuits in the LgDel mouse model of 22q11.2DS	2015	Cerebral Cortex	25	1143	1151	T&A	Excluded	no human
**Demily C., Franck N.**	Cognitive behavioral therapy in 22q11.2 microdeletion with psychotic symptoms: What do we learn from schizophrenia?	2016	European Journal of Medical Genetics	59	596	603	T&A	Excluded	wrong study design
**De Smedt B., Swillen A., Devriendt K., Fryns J.-P., Verschaffel L., Boets B., Ghesquière P.**	Cognitive correlates of mathematical disabilities in children with velo-cardio-facial syndrome	2008	Genetic Counseling	19	71	94	T&A	Excluded	wrong outcome
**Keefe R.S.E., Kahn R.S.**	Cognitive decline and disrupted cognitive trajectory in schizophrenia	2017	JAMA Psychiatry	74	535	536	T&A	Excluded	wrong topic
**Biswas A.B., Furniss F.**	Cognitive phenotype and psychiatric disorder in 22q11.2 deletion syndrome: A review	2016	Research in Developmental Disabilities	53-54	242	257	T&A	Excluded	wrong study design
**Mariano M.A., Tang K., Kurtz M., Kates W.R.**	Cognitive remediation for adolescents with 22q11 deletion syndrome (22q11DS): A preliminary study examining effectiveness, feasibility, and fidelity of a hybrid strategy, remote and computer-based intervention	2015	Schizophrenia Research	166	283	289	T&A	Excluded	wrong outcome
**Rigard C., Peyroux E., Morel A., Demily C.**	Cognitive remediation in child psychiatry [Remédiation cognitive en psychiatrie de l'enfant]	2016	ANAE - Approche Neuropsychologique des Apprentissages chez l'Enfant	28	233	240	T&A	Excluded	wrong topic
**Manolakos E., Sarri C., Vetro A., Kefalas K., Leze E., Sofocleus C., Kitsos G., Merou K., Kokotas H., Papadopoulou A., Attilakos A., Petersen M.B., Kitsiou-Tzeli S.**	Combined 22q11.1-q11.21 deletion with 15q11.2-q13.3 duplication identified by array-CGH in a 6 years old boy	2011	Molecular Cytogenetics	4			T&A	Excluded	wrong study design

**Fernández L., Lapunzina P., Arjona D., López Pajares I., García-Guereta L., Elorza D., Burgueros M., De Torres M.L., Mori M.A., Palomares M., García-Alix A., Delicado A.**	Comparative study of three diagnostic approaches (FISH, STRs and MLPA) in 30 patients with 22q11.2 deletion syndrome	2005	Clinical Genetics	68	373	378	FT	Excluded	incomplete outcome
**Schneider M., Debbané M., Lagioia A., Salomon R., D'Argembeau A., Eliez S.**	Comparing the neural bases of self-referential processing in typically developing and 22q11.2 adolescents	2012	Developmental Cognitive Neuroscience	2	277	289	T&A	Excluded	wrong outcome
**Hunsaker M.R.**	Comprehensive neurocognitive endophenotyping strategies for mouse models of genetic disorders	2012	Progress in Neurobiology	96	220	241	T&A	Excluded	no human
**Chen Y.-F., Kou P.-L., Tsai S.-J., Chen K.-F., Chan H.-H., Chen C.-M., Sun H.S.**	Computational analysis and refinement of sequence structure on chromosome 22q11.2 region: Application to the development of quantitative real-time PCR assay for clinical diagnosis	2006	Genomics	87	290	297	T&A	Excluded	wrong outcome
**Gothelf D., Eliez S., Thompson T., Hinard C., Penniman L., Feinstein C., Kwon H., Jin S., Jo B., Antonarakis S.E., Morris M.A., Reiss A.L.**	COMT genotype predicts longitudinal cognitive decline and psychosis in 22q11.2 deletion syndrome	2005	Nature Neuroscience	8	1500	1502	T&A	Excluded	wrong outcome
**Blennow E., Lagerstedt K., Malmgren S., Sahlén S., Schoumans J., Anderlid B.M.**	Concurrent microdeletion and duplication of 22q11.2	2008	Clinical Genetics	74	61	67	T&A	Excluded	wrong topic
**Hakim Z.S., DiMichele L.A., Doherty J.T., Homeister J.W., Beggs H.E., Reichardt L.F., Schwartz R.J., Brackhan J., Smithies O., Mack C.P., Taylor J.M.**	Conditional deletion of focal adhesion kinase leads to defects in ventricular septation and outflow tract alignment	2007	Molecular and Cellular Biology	27	5352	5364	T&A	Excluded	no human
**Goodman F.R.**	Congenital abnormalities of body patterning: Embryology revisited	2003	Lancet	362	651	662	T&A	Excluded	wrong topic

**Marino B., Mileto F., Digilio M.C., Carotti A., Di Donato R.**	Congenital cardiovascular disease and velo-cardio-facial syndrome	2005	Velo-Cardio-Facial Syndrome: A Model for Understanding Microdeletion Disorders		47	82	T&A	Excluded	wrong study design
**Matthiesen N.B., Agergaard P., Henriksen T.B., Bach C.C., Gaynor J.W., Hjortdal V., Østergaard J.R.**	Congenital Heart Defects and Measures of Fetal Growth in Newborns with Down Syndrome or 22q11.2 Deletion Syndrome	2016	Journal of Pediatrics	175	116	122	T&A	Excluded	wrong outcome
**Schaer M., Glaser B., Cuadra M.B., Debbane M., Thiran J.-P., Eliez S.**	Congenital heart disease affects local gyrification in 22q11.2 deletion syndrome	2009	Developmental Medicine and Child Neurology	51	746	753	T&A	Excluded	wrong outcome
**Fountain D.M., Schaer M., Mutlu A.K., Schneider M., Debbané M., Eliez S.**	Congenital heart disease is associated with reduced cortical and hippocampal volume in patients with 22q11.2 deletion syndrome	2014	Cortex	57	128	142	T&A	Excluded	wrong outcome
**Kunishima S., Saito H.**	Congenital macrothrombocytopenias	2006	Blood Reviews	20	111	121	T&A	Excluded	wrong topic
**Jobe S.M.**	Congenital Thrombocytopenia	2009	Transfusion Medicine and Hemostasis		445	451	T&A	Excluded	wrong topic
**Karunamuni G.H., Ma P., Gu S., Rollins A.M., Jenkins M.W., Watanabe M.**	Connecting teratogen-induced congenital heart defects to neural crest cells and their effect on cardiac function	2014	Birth Defects Research Part C - Embryo Today: Reviews	102	227	250	T&A	Excluded	wrong topic
**Machado A.M.C., Teixeira C.A.C.**	Content-based retrieval of medical images with elongated structures	2008	Communications in Computer and Information Science	25 CC	189	201	T&A	Excluded	wrong topic
**Schreiner M.J., Lazaro M.T., Jalbrzikowski M., Bearden C.E.**	Converging levels of analysis on a genomic hotspot for psychosis: Insights from 22q11.2 Deletion Syndrome	2013	Neuropharmacology	68	157	173	T&A	Excluded	wrong study design
**D'Angelo C.S., Koiffmann C.P.**	Copy number variants in obesity-related syndromes: Review and perspectives on novel molecular approaches	2012	Journal of Obesity				T&A	Excluded	wrong topic

**Levinson D.F., Duan J., Oh S., Wang K., Sanders A.R., Shi J., Zhang N., Mowry B.J., Olincy A., Amin F., Cloninger C.R., Silverman J.M., Buccola N.G., Byerley W.F., Black D.W., Kendler K.S., Freedman R., Dudbridge F., Pe'er I., Hakonarson H., Bergen S.E., Fanous A.H., Holmans P.A., Gejman P.V.**	Copy number variants in schizophrenia: Confirmation of five previous finding sand new evidence for 3q29 microdeletions and VIPR2 duplications	2011	American Journal of Psychiatry	168	302	316	T&A	Excluded	wrong topic
**Wit J.M., Van Duyvenvoorde H.A., Van Klinken J.B., Caliebe J., Bosch C.A.J., Lui J.C., Gijsbers A.C.J., Bakker E., Breuning M.H., Oostdijk W., Losekoot M., Baron J., Binder G., Ranke M.B., Ruivenkamp C.A.L.**	Copy number variants in short children born small for gestational age	2014	Hormone Research in Paediatrics	82	310	318	T&A	Excluded	wrong topic
**Breckpot J., Vercruyssen M., Weyts E., Vandevoort S., D'Haenens G., Van Buggenhout G., Leempoels L., Brischoux-Boucher E., Van Maldergem L., Renieri A., Mencarelli M.A., D'Angelo C., Mericq V., Hoffer M.J., Tauber M., Molinas C., Castiglioni C., Brison N., Vermeesch J.R., Danckaerts M., Sienaert P., Devriendt K., Vogels A.**	Copy number variation analysis in adults with catatonia confirms haploinsufficiency of SHANK3 as a predisposing factor	2016	European Journal of Medical Genetics	59	436	443	T&A	Excluded	wrong topic
**Szatkiewicz J.P., O'Dushlaine C., Chen G., Chambert K., Moran J.L., Neale B.M., Fromer M., Ruderfer D., Akterin S., Bergen S.E., Kähler A., Magnusson P.K.E., Kim Y., Crowley J.J., Rees E., Kirov G., O'Donovan M.C., Owen M.J., Walters J., Scolnick E., Sklar P., Purcell S., Hultman C.M., McCarroll S.A., Sullivan P.F.**	Copy number variation in schizophrenia in Sweden	2014	Molecular Psychiatry	19	762	773	FT	Excluded	incomplete outcome
**Grayton H.M., Fernandes C., Rujescu D., Collier D.A.**	Copy number variations in neurodevelopmental disorders	2012	Progress in Neurobiology	99	81	91	T&A	Excluded	wrong topic

**Mlynarski E.E., Sheridan M.B., Xie M., Guo T., Racedo S.E., McDonald-Mcginn D.M., Gai X., Chow E.W.C., Vorstman J., Swillen A., Devriendt K., Breckpot J., Digilio M.C., Marino B., Dallapiccola B., Philip N., Simon T.J., Roberts A.E., Piotrowicz M., Bearden C.E., Eliez S., Gothelf D., Coleman K., Kates W.R., Devoto M., Zackai E., Heine-Suñer D., Shaikh T.H., Bassett A.S., Goldmuntz E., Morrow B.E., Emanuel B.S.**	Copy-number variation of the glucose transporter gene SLC2A3 and congenital heart defects in the 22q11.2 deletion syndrome	2015	American Journal of Human Genetics	96	753	764	FT	Included	
**Machado A.M.C., Simon T.J., Nguyen V., McDonald-McGinn D.M., Zackai E.H., Gee J.C.**	Corpus callosum morphology and ventricular size in chromosome 22q11.2 deletion syndrome	2007	Brain Research	1131	197	210	T&A	Excluded	wrong outcome
**Matsuoka R., Shimizu N., Kimura M., Minoshima S., Mamura S.-I., Takao A., Momma K.**	Correlation between smaller deletion size and mental retardation in de122q11.2 syndrome	1997	Japanese Journal of Human Genetics	42	122		T&A	Excluded	not available
**Flore G., Cioffi S., Bilio M., Illingworth E.**	Cortical Development Requires Mesodermal Expression of Tbx1, a Gene Haploinsufficient in 22q11.2 Deletion Syndrome	2017	Cerebral cortex (New York, N.Y. : 1991)	27	2210	2225	T&A	Excluded	no human
**Kunwar A., Ramanathan S., Nelson J., Antshel K.M., Fremont W., Higgins A.M., Shprintzen R.J., Kates W.R.**	Cortical gyrification in velo-cardio-facial (22q11.2 deletion) syndrome: A longitudinal study	2012	Schizophrenia Research	137	20	25	T&A	Excluded	wrong outcome
**Berhanu D., Mattiaccio L.M., Antshel K.M., Fremont W., Middleton F.A., Kates W.R.**	Cortical-amygdala volumetric ratios predict onset of symptoms of psychosis in 22q11.2 deletion syndrome	2017	Psychiatry Research - Neuroimaging	259	10	15	T&A	Excluded	wrong outcome
**Cordero D.R., Brugmann S., Chu Y., Bajpai R., Jame M., Helms J.A.**	Cranial neural crest cells on the move: Their roles in craniofacial development	2011	American Journal of Medical Genetics, Part A	155	270	279	T&A	Excluded	wrong study design

**Clements C.C., Wenger T.L., Zoltowski A.R., Bertollo J.R., Miller J.S., De Marchena A.B., Mitteer L.M., Carey J.C., Yerys B.E., Zackai E.H., Emanuel B.S., McDonald-McGinn D.M., Schultz R.T.**	Critical region within 22q11.2 linked to higher rate of autism spectrum disorder	2017	Molecular Autism	8			FT	Excluded	incomplete outcome
**Yaron Y., Jani J., Schmid M., Oepkes D.**	Current status of testing for microdeletion syndromes and rare autosomal trisomies using cell-free DNA technology	2015	Obstetrics and Gynecology	126	1095	1099	T&A	Excluded	wrong topic
**García G.A.G., Vargas C.M.T., Samudio C.G.G., Peña C.M.M., Arango J.C.O., Restrepo J.L.F.**	Cytogenetic evaluation and loss of heterozigocity of chromosome 22q11.2 in patients with the DiGeorge syndrome [Evaluación citogenética y de pérdida de la heterocigosidad de la región 22q11.2 en pacientes con el síndrome de DiGeorge]	2011	Iatreia	24	229	237	T&A	Excluded	wrong study design
**Feenstra I., Brunner H.G., Van Ravenswaaij C.M.A.**	Cytogenetic genotype-phenotype studies: Improving genotyping, phenotyping and data storage	2006	Cytogenetic and Genome Research	115	231	239	T&A	Excluded	wrong topic
**Sellier C., Hwang V.J., Dandekar R., Durbin-Johnson B., Charlet-Berguerand N., Ander B.P., Sharp F.R., Angkustsiri K., Simon T.J., Tassone F.**	Decreased DGCR8 expression and miRNA dysregulation in individuals with 22q11.2 deletion syndrome	2014	PLoS ONE	9			T&A	Excluded	wrong outcome
**Markert M.L.**	Defects in Thymic Development: DiGeorge/CHARGE/Chromosome 22q11.2 Deletion	2014	Stiehm's Immune Deficiencies		221	242	T&A	Excluded	wrong publication type
**Monteiro F.P., Vieira T.P., Sgardioli I.C., Molck M.C., Damiano A.P., Souza J., Monlleó I.L., Fontes M.I.B., Fett-Conte A.C., Félix T.M., Leal G.F., M.ribeiro E., Banzato C.E.M., Dantas C.D.R., Lopes-Cendes I., Gil-Da-Silva-Lopes V.L.**	Defining new guidelines for screening the 22q11.2 deletion based on a clinical and dysmorphologic evaluation of 194 individuals and review of the literature	2013	European Journal of Pediatrics	172	927	945	FT	Included	

**Hay B.N.**	Deletion 22q11: Spectrum of Associated Disorders	2007	Seminars in Pediatric Neurology	14	136	139	T&A	Excluded	wrong study design
**Glausier J.R., Lewis D.A.**	Dendritic spine pathology in schizophrenia	2013	Neuroscience	251	90	107	T&A	Excluded	wrong topic
**Martín B., Fañanás L., Gutiérrez B., Chow E.W.C., Bassett A.S.**	Dermatoglyphic profile in 22q Deletion syndrome	2004	American Journal of Medical Genetics - Neuropsychiatric Genetics	128 B	46	49	T&A	Excluded	wrong outcome
**Zhao C., Tynan J., Ehrich M., Hannum G., McCullough R., Saldivar J.-S., Oeth P., Van Den Boom D., Deciu C.**	Detection of fetal subchromosomal abnormalities by sequencing circulating cell-free DNA from maternal plasma	2015	Clinical Chemistry	61	608	616	T&A	Excluded	wrong topic
**Hrusca A., Rachisan A.L., Gach P., Pico H., Sorensen C., Bonello B., Ovaert C., Petit P., Fouilloux V., Mace L., Gorincour G.**	Detection of pulmonary and coronary artery anomalies in tetralogy of Fallot using non-ECG-gated CT angiography	2016	Diagnostic and Interventional Imaging	97	543	548	T&A	Excluded	wrong topic
**Tan T.Y., Kilpatrick N., Farlie P.G.**	Developmental and genetic perspectives on pierre robin sequence	2013	American Journal of Medical Genetics, Part C: Seminars in Medical Genetics	163	295	305	T&A	Excluded	wrong topic
**Paul L.K.**	Developmental malformation of the corpus callosum: A review of typical callosal development and examples of developmental disorders with callosal involvement	2011	Journal of Neurodevelopmenta l Disorders	3	3	27	T&A	Excluded	wrong topic
**Gothelf D., Penniman L., Gu E., Eliez S., Reiss A.L.**	Developmental trajectories of brain structure in adolescents with 22q11.2 deletion syndrome: A longitudinal study	2007	Schizophrenia Research	96	72	81	T&A	Excluded	wrong outcome
**Schaer M., Debbané M., Bach Cuadra M., Ottet M.-C., Glaser B., Thiran J.-P., Eliez S.**	Deviant trajectories of cortical maturation in 22q11.2 deletion syndrome (22q11DS): A cross-sectional and longitudinal study	2009	Schizophrenia Research	115	182	190	T&A	Excluded	wrong outcome

**Jamieson A., Smith C.J.**	Dilated cardiomyopathy: A preventable presentation of DiGeorge syndrome	2015	Journal of the Royal College of Physicians of Edinburgh	45	273	275	T&A	Excluded	wrong study design
**Chaoui R., Heling K.-S., Zhao Y., Sinkovskaya E., Abuhamad A., Karl K.**	Dilated cavum septi pellucidi in fetuses with microdeletion 22q11	2016	Prenatal Diagnosis	36	911	915	T&A	Excluded	wrong outcome
**Schmitt J.E., Yi J., Calkins M.E., Ruparel K., Roalf D.R., Cassidy A., Souders M.C., Satterthwaite T.D., McDonald-McGinn D.M., Zackai E.H., Gur R.C., Emanuel B.S., Gur R.E.**	Disrupted anatomic networks in the 22q11.2 deletion syndrome	2016	NeuroImage: Clinical	12	420	428	T&A	Excluded	wrong outcome
**Deng Y., Goodrich-Hunsaker N.J., Cabaral M., Amaral D.G., Buonocore M.H., Harvey D., Kalish K., Carmichael O.T., Schumann C.M., Lee A., Dougherty R.F., Perry L.M., Wandell B.A., Simon T.J.**	Disrupted fornix integrity in children with chromosome 22q11.2 deletion syndrome	2015	Psychiatry Research - Neuroimaging	232	106	114	T&A	Excluded	wrong outcome
**Classen C.F., Riehmer V., Landwehr C., Kosfeld A., Heilmann S., Scholz C., Kabisch S., Engels H., Tierling S., Zivicnjak M., Schacherer F., Haffner D., Weber R.G.**	Dissecting the genotype in syndromic intellectual disability using whole exome sequencing in addition to genome-wide copy number analysis	2013	Human Genetics	132	825	841	T&A	Excluded	wrong topic
**Chen H.-I., Chien Y.-L., Liao H.-M., Chien W.-H., Chen C.-H., Chen Y.-C., Gau S.S.F.**	Dosage of copy number variation at 22q11.2 mediates changes in cognition, social function and brain structure in autism spectrum disorder	2016	Journal of the Formosan Medical Association	115	577	582	T&A	Excluded	wrong outcome
**Lania G., Zhang Z., Huynh T., Caprio C., Moon A.M., Vitelli F., Baldini A.**	Early thyroid development requires a Tbx1-Fgf8 pathway	2009	Developmental Biology	328	109	117	T&A	Excluded	no human
**Ushiroda J., Inoue S., Furuya H., Kawaguchi M.**	Effective use of laryngeal mask airway to stop a supraglottic air leak which prevented adequate ventilation via a tracheostomy in a pediatric patient	2013	Japanese Journal of Anesthesiology	62	1360	1361	T&A	Excluded	wrong topic

**Van Amelsvoort T., Zinkstok J., Figee M., Daly E., Morris R., Owen M.J., Murphy K.C., De Haan L., Linszen D.H., Glaser B., Murphy D.G.M.**	Effects of a functional COMT polymorphism on brain anatomy and cognitive function in adults with velo-cardio-facial syndrome	2008	Psychological Medicine	38	89	100	T&A	Excluded	wrong outcome
**Edelmann L., Funke B., Pandita R.K., Puech A., Saint-Lore B., Goldberg R., Shprintzen R., Kucherlapati R., Skoultch A., Morrow B.E.**	Efforts to determine the molecular basis of VCFS	1999	Genetic Counseling	10	106		T&A	Excluded	not available
**Zhou J., Pashmforoush M., Sucov H.M.**	Endothelial neuropilin disruption in mice causes digeorge syndrome-like malformations via mechanisms distinct to those caused by loss of tbx1	2012	PLoS ONE	7			T&A	Excluded	no human
**Delio M., Guo T., McDonald-Mcginn D.M., Zackai E., Herman S., Kaminetzky M., Higgins A.M., Coleman K., Chow C., Jarlbrzkowski M., Bearden C.E., Bailey A., Vangkilde A., Olsen L., Olesen C., Skovby F., Werge T.M., Templin L., Busa T., Philip N., Swillen A., Vermeesch J.R., Devriendt K., Schneider M., Dahoun S., Eliez S., Schoch K., Hooper S.R., Shashi V., Samanich J., Marion R., Van Amelsvoort T., Boot E., Klaassen P., Duijff S.N., Vorstman J., Yuen T., Silversides C., Chow E., Bassett A., Frisch A., Weizman A., Gothelf D., Niarchou M., Van Den Bree M., Owen M.J., Suñer D.H., Andreo J.R., Armando M., Vicari S., Digilio M.C., Auton A., Kates W.R., Wang T., Shprintzen R.J., Emanuel B.S., Morrow B.E.**	Enhanced maternal origin of the 22q11.2 deletion in velocardiofacial and digeorge syndromes	2013	American Journal of Human Genetics	92	439	447	T&A	Excluded	wrong outcome
**Bingham P.M., Zimmerman R.A., McDonald-McGinn D., Driscoll D., Emanuel B.S., Zackai E.**	Enlarged Sylvian fissures in infants with interstitial deletion of chromosome 22q11	1997	American Journal of Medical Genetics - Neuropsychiatric Genetics	74	538	543	T&A	Excluded	wrong outcome

**Wieczorek D., Shaw-Smith C., Kohlhase J., Schmitt W., Buiting K., Coffey A., Howard E., Hehr U., Gillessen-Kaesbach G.**	Esophageal atresia, hypoplasia of zygomatic complex, microcephaly, cup-shaped ears, congenital heart defect, and mental retardation - New MCA/MR syndrome in two affected sibs and a mildly affected mother?	2007	American Journal of Medical Genetics, Part A	143	1135	1142	T&A	Excluded	wrong topic
**Betancur C.**	Etiological heterogeneity in autism spectrum disorders: More than 100 genetic and genomic disorders and still counting	2011	Brain Research	1380	42	77	T&A	Excluded	wrong topic
**Deng J.-Y., Zhang Z.-W., Li J.-H., Zhu Y.-N., Yang J.-B., Gao Z., Ying L.-Y.**	Evaluation of detection and analysis of chromosome 22q11. 2 microdeletion by multiple ligation-dependent probe amplification assay	2011	Chinese Journal of Medical Genetics	28	190	194	T&A	Excluded	foreing lenguage
**Stoll C., Garne E., Clementi M.**	Evaluation of prenatal diagnosis of associated congenital heart diseases by fetal ultrasonographic examination in Europe	2001	Prenatal Diagnosis	21	243	252	T&A	Excluded	wrong topic
**Shashi V., Kwapil T.R., Kaczorowski J., Berry M.N., Santos C.S., Howard T.D., Goradia D., Prasad K., Vaibhav D., Rajarethinam R., Spence E., Keshavan M.S.**	Evidence of gray matter reduction and dysfunction in chromosome 22q11.2 deletion syndrome	2010	Psychiatry Research - Neuroimaging	181	1	8	T&A	Excluded	wrong outcome
**Wapner R.J., Babiarz J.E., Levy B., Stosic M., Zimmermann B., Sigurjonsson S., Wayham N., Ryan A., Banjevic M., Lacroute P., Hu J., Hall M.P., Demko Z., Siddiqui A., Rabinowitz M., Gross S.J., Hill M., Benn P.**	Expanding the scope of noninvasive prenatal testing: Detection of fetal microdeletion syndromes	2015	American Journal of Obstetrics and Gynecology	212	3320	3320	T&A	Excluded	wrong topic

**Klaassen P., Duijff S., Swanenburg de Veye H., Beemer F., Sinnema G., Breetvelt E., Schappin R., Vorstman J.**	Explaining the variable penetrance of CNVs: Parental intelligence modulates expression of intellectual impairment caused by the 22q11.2 deletion	2016	American Journal of Medical Genetics, Part B: Neuropsychiatric Genetics	171	790	796	T&A	Excluded	wrong outcome
**Spruijt N.E., Sameer Rana M.S., Christoffels V.M., Mink van der Molen A.B.**	Exploring a neurogenic basis of velopharyngeal dysfunction in Tbx1 mutant mice: No difference in volumes of the nucleus ambiguus	2013	International Journal of Pediatric Otorhinolaryngolog y	77	1002	1007	T&A	Excluded	no human
**Zhou H., Perkins S.L., Tripp S., Hussong J.W., Coffin C.M.**	Expression of apoptosis-related antigens, Fas, bcl-2, p53, and Mib-1 proliferation index in the hypoplastic thymus of DiGeorge syndrome	2002	Pediatric and Developmental Pathology	5	465	471	T&A	Excluded	wrong outcome
**Markert M.L., Devlin B.H., Chinn I.K., McCarthy E.A., Li Y.J.**	Factors affecting success of thymus transplantation for complete DiGeorge anomaly	2008	American Journal of Transplantation	8	1729	1736	T&A	Excluded	wrong outcome
**Adeyinka A., Stockero K.J., Flynn H.C., Lorentz C.P., Ketterling R.P., Jalal S.M.**	Familial 22q11.2 deletions in DiGeorge/velocardiofacial syndrome are predominantly smaller than the commonly observed 3Mb	2004	Genetics in Medicine	6	517	520	FT	Excluded	incomplete outcome
**Harrell W., Eack S., Hooper S.R., Keshavan M.S., Bonner M.S., Schoch K., Shashi V.**	Feasibility and preliminary efficacy data from a computerized cognitive intervention in children with chromosome 22q11.2 deletion syndrome	2013	Research in Developmental Disabilities	34	2606	2613	T&A	Excluded	wrong outcome
**Zhao Y., Edington S., Fleenor J., Sinkovskaya E., Porche L., Abuhamad A.**	Fetal cardiac axis in tetralogy of Fallot: associations with prenatal findings, genetic anomalies and postnatal outcome	2017	Ultrasound in Obstetrics and Gynecology	50	58	62	T&A	Excluded	wrong topic
**Van L., Butcher N.J., Costain G., Ogura L., Chow E.W.C., Bassett A.S.**	Fetal growth and gestational factors as predictors of schizophrenia in 22q11.2 deletion syndrome	2016	Genetics in Medicine	18	350	355	T&A	Excluded	wrong outcome

**D'Antonio F., Khalil A., Zidere V., Carvalho J.S.**	Fetuses with right aortic arch: A multicenter cohort study and meta-analysis	2016	Ultrasound in Obstetrics and Gynecology	47	423	432	T&A	Excluded	wrong topic
**Bartsch O., Rasi S., Hoffmann K., Blin N.**	FISH of supernumerary marker chromosomes (SMCs) identifies six diagnostically relevant intervals on chromosome 22q and a novel type of bisatellited SMC (22)	2005	European Journal of Human Genetics	13	592	598	T&A	Excluded	wrong topic
**Mclean-Tooke A., Barge D., Spickett G.P., Gennery A.R.**	Flow Cytometric Analysis of TCR Vβ Repertoire in Patients with 22q11.2 Deletion Syndrome	2011	Scandinavian Journal of Immunology	73	577	585	T&A	Excluded	wrong outcome
**Latger-Cannard V., Bensoussan D., Grégoire M.-J., Marcon F., Cloez J.-L., Leheup B., Jonveaux P., Lecompte T., Bordigoni P.**	Frequency of thrombocytopenia and large platelets correlates neither with conotruncal cardiac anomalies nor immunological features in the chromosome 22q11.2 deletion syndrome	2004	European Journal of Pediatrics	163	327	328	T&A	Excluded	wrong outcome
**Schaer M., Eliez S.**	From Genes to Brain: Understanding Brain Development in Neurogenetic Disorders Using Neuroimaging Techniques	2007	Child and Adolescent Psychiatric Clinics of North America	16	557	579	T&A	Excluded	wrong topic
**Kates W.R., Burnette C.P., Bessette B.A., Folley B.S., Strunge L., Jabs E., Pearlson G.D.**	Frontal and caudate alterations in velocardiofacial syndrome (deletion at chromosome 22q11.2)	2004	Journal of Child Neurology	19	337	342	T&A	Excluded	wrong outcome
**Harrell W., Zou L., Englander Z., Hooper S.R., Keshavan M.S., Song A., Shashi V.**	Frontal Hypoactivation during a Working Memory Task in Children with 22q11 Deletion Syndrome	2017	Journal of Child Neurology	32	94	99	T&A	Excluded	wrong outcome
**Kempf L., Nicodemus K.K., Kolachana B., Vakkalanka R., Verchinski B.A., Egan M.F., Straub R.E., Mattay V.A., Callicott J.H., Weinberger D.R., Meyer-Lindenberg A.**	Functional polymorphisms in PRODH are associated with risk and protection for schizophrenia and fronto-striatal structure and function	2008	PLoS Genetics	4			T&A	Excluded	wrong outcome

**Lindquist S.G., Kirchhoff M., Lundsteen C., Pedersen W., Erichsen G., Kristensen K., Lillquist K., Smedegaard H.H., Skov L., Tommerup N., Brøndum-Nielsen K.**	Further delineation of the 22q13 deletion syndrome	2005	Clinical Dysmorphology	14	55	60	T&A	Excluded	wrong topic
**Ferreira L.M.R.**	Gammadelta T Cells: Innately adaptive immune cells	2013	International Reviews of Immunology	32	223	248	T&A	Excluded	wrong topic
**Kates W.R., Antshel K., Willhite R., Bessette B.A., AbdulSabur N., Higgins A.M.**	Gender-moderated dorsolateral prefrontal reductions in 22q11.2 Deletion Syndrome: Implications for risk for schizophrenia	2005	Child Neuropsychology	11	73	85	T&A	Excluded	wrong outcome
**Kwiatkowska J., Wierzba J., Aleszewicz-Baranowska J., Ereciński J.**	Genetic background of congenital conotruncal heart defects - A study of 45 families	2007	Kardiologia Polska	65	32	37	FT	Excluded	incomplete outcome
**Baker K., Raymond F.L., Bass N.**	Genetic investigation for adults with intellectual disability: Opportunities and challenges	2012	Current Opinion in Neurology	25	150	158	T&A	Excluded	wrong topic
**Berdasco M., Esteller M.**	Genetic syndromes caused by mutations in epigenetic genes	2013	Human Genetics	132	359	383	T&A	Excluded	wrong topic
**Muir W.J.**	Genetics advances and learning disability	2000	British Journal of Psychiatry	176	12	19	T&A	Excluded	wrong topic
**Llevadot R., Scambler P., Estivill X., Pritchard M.**	Genomic organization of TUPLE1/HIRA: A gene implicated in DiGeorge syndrome	1996	Mammalian Genome	7	911	914	T&A	Excluded	no human

**Guo T., Mcdonald-Mcginn D., Blonska A., Shanske A., Bassett A.S., Chow E., Bowser M., Sheridan M., Beemer F., Devriendt K., Swillen A., Breckpot J., Digilio M.C., Marino B., Dallapiccola B., Carpenter C., Zheng X., Johnson J., Chung J., Higgins A.M., Philip N., Simon T.J., Coleman K., Heine-Suner D., Rosell J., Kates W., Devoto M., Goldmuntz E., Zackai E., Wang T., Shprintzen R., Emanuel B., Morrow B.**	Genotype and cardiovascular phenotype correlations with TBX1 in 1,022 velo-cardio-facial/digeorge/22q11.2 deletion syndrome patients	2011	Human Mutation	32	1278	1289	T&A	Excluded	wrong outcome
**Michaelovsky E., Frisch A., Carmel M., Patya M., Zarchi O., Green T., Basel-Vanagaite L., Weizman A., Gothelf D.**	Genotype-phenotype correlation in 22q11.2 deletion syndrome	2012	BMC Medical Genetics	13			FT	Included	
**Eyske B., Devriendt S.K., Vermylrn J., Hoylaerts M.K., Fryns J.P., Gewillig M., Van Geet C.**	Giant platelets in velo-cardio-facial syndrome	1999	Genetic Counseling	10	113		T&A	Excluded	wrong outcome
**Saint-Jore B., Puech A., Heyer J., Lin Q., Raine C., Kucherlapati R., Skoultchi A.I.**	Goosecoid-like (Gscl), a candidate gene for velocardiofacial syndrome, is not essential for normal mouse development	1998	Human Molecular Genetics	7	1841	1849	T&A	Excluded	no human
**Ottet M.-C., Schaer M., Debbané M., Cammoun L., Thiran J.-P., Eliez S.**	Graph theory reveals dysconnected hubs in 22q11DS and altered nodal efficiency in patients with hallucinations	2012	Frontiers in Human Neuroscience				T&A	Excluded	wrong outcome
**O'Driscoll M.**	Haploinsufficiency of DNA damage response genes and their potential influence in human genomic disorders	2008	Current Genomics	9	137	146	T&A	Excluded	wrong topic
**Devaraju P., Yu J., Eddins D., Mellado-Lagarde M.M., Earls L.R., Westmoreland J.J., Quarato G., Green D.R., Zakharenko S.S.**	Haploinsufficiency of the 22q11.2 microdeletion gene Mrpl40 disrupts short-term synaptic plasticity and working memory through dysregulation of mitochondrial calcium	2017	Molecular Psychiatry	22	1313	1326	T&A	Excluded	no human

**Wright D.T., Nguyen S.A., Teufel R.J., II, White D.R.**	Health care resource use in patients with and without 22q11.2 deletion syndrome undergoing sphincter pharyngoplasty for velopharyngeal insufficiency	2017	JAMA Otolaryngology - Head and Neck Surgery	143	286	291	T&A	Excluded	wrong outcome
**Medelros P.M., Alembik Y., De Cekter B., Livolai A., Soskin S., Gilgenkrantz S., Stoll C.**	Heterogeneity of clinical features in catch22 syndrome	1999	Genetic Counseling	10	99		T&A	Excluded	not available
**Liang H.P.H., Morel-Kopp M.-C., Curtin J., Wilson M., Hewson J., Chen W., Ward C.M.**	Heterozygous loss of platelet glycoprotein (GP) Ib-V-IX variably affects platelet function in velocardiofacial syndrome (VCFS) patients	2007	Thrombosis and Haemostasis	98	1298	1308	T&A	Excluded	wrong outcome
**Nik-Zainal S., Strick R., Storer M., Huang N., Rad R., Willatt L., Fitzgerald T., Martin V., Sandford R., Carter N.P., Janecke A.R., Renner S.P., Oppelt P.G., Oppelt P., Schulze C., Brucker S., Hurles M., Beckmann M.W., Strissel P.L., Shaw-Smith C.**	High incidence of recurrent copy number variants in patients with isolated and syndromic Müllerian aplasia	2011	Journal of Medical Genetics	48	197	204	T&A	Excluded	wrong topic
**Fernández L., Lapunzina P., López Pajares I., Rodríguez Criado G., García-Guereta L., Pérez J., Quero J., Delicado A.**	Higher frequency of uncommon 1.5-2 Mb deletions found in familial cases of 22q11.2 deletion syndrome	2005	American Journal of Medical Genetics	136 A	71	75	FT	Included	
**Debbané M., Schaer M., Farhoumand R., Glaser B., Eliez S.**	Hippocampal volume reduction in 22q11.2 deletion syndrome	2006	Neuropsychologia	44	2360	2365	T&A	Excluded	wrong outcome
**DeBoer T., Wu Z., Lee A., Simon T.J.**	Hippocampal volume reduction in children with chromosome 22q11.2 deletion syndrome is associated with cognitive impairment	2007	Behavioral and Brain Functions	3			T&A	Excluded	wrong outcome

**Flahault A., Schaer M., Ottet M.-C., Debbané M., Eliez S.**	Hippocampal volume reduction in chromosome 22q11.2 deletion syndrome (22q11.2DS): A longitudinal study of morphometry and symptomatology	2012	Psychiatry Research - Neuroimaging	203	1	5	T&A	Excluded	wrong outcome
**Widdershoven J.C.C., Spruijt N.E., Spliet W.G.M., Breugem C.C., Kon M., van der Molen A.B.M.**	Histology of the pharyngeal constrictor muscle in 22q11.2 deletion syndrome and non-syndromic children with velopharyngeal insufficiency	2011	PLoS ONE	6			T&A	Excluded	wrong outcome
**Hudson L.L., Louise Markert M., Devlin B.H., Haynes B.F., Sempowski G.D.**	Human T cell reconstitution in DiGeorge syndrome and HIV-1 infection	2007	Seminars in Immunology	19	297	309	T&A	Excluded	wrong study design
**Raghavan V., Bhomia M., Torres I., Jain S., Wang K.K.**	Hypothesis: Exosomal microRNAs as potential biomarkers for schizophrenia	2017	Medical Hypotheses	103	21	25	T&A	Excluded	wrong topic
**Jawad A.F., McDonald-McGinn D.M., Zackai E., Sullivan K.E.**	Immunologic features of chromosome 22q11.2 deletion syndrome (DiGeorge syndrome/velocardiofacial syndrome)	2001	Journal of Pediatrics	139	715	723	T&A	Excluded	wrong outcome
**Lazar D.A., Cass D.L., Rodriguez M.A., Hassan S.F., Cassady C.I., Johnson Y.R., Johnson K.E., Johnson A., Moise K.J., Belleza-Bascon B., Olutoye O.O.**	Impact of prenatal evaluation and protocol-based perinatal management on congenital diaphragmatic hernia outcomes	2011	Journal of Pediatric Surgery	46	808	813	T&A	Excluded	wrong topic
**Cuturilo G., Drakulic D., Jovanovic I., Krstic A., Djukic M., Skoric D., Mijovic M., Stefanovic I., Milivojevic M., Stevanovic M.**	Improving the diagnosis of children with 22q11.2 deletion syndrome: A single-center experience from Serbia	2016	Indian Pediatrics	53	786	789	FT	Excluded	incomplete outcome
**Rauch A., Hofbeck M., Leipold G., Klinge J., Trautmann U., Kirsch M., Singer H., Pfeiffer R.A.**	Incidence and significance of 22q11.2 hemizygosity in patients with interrupted aortic arch	1998	American Journal of Medical Genetics	78	322	331	FT	Excluded	incomplete outcome
**Maisenbacher M.K., Merrion K., Pettersen B., Young M., Paik K., Iyengar S., Kareht S., Sigurjonsson S., Demko Z.P., Martin K.A.**	Incidence of the 22q11.2 deletion in a large cohort of miscarriage samples	2017	Molecular Cytogenetics	10			T&A	Excluded	wrong outcome

**Schmitt J.E., Yi J.J., Roalf D.R., Loevner L.A., Ruparel K., Whinna D., Souders M.C., Mcdonald-Mcginn D.M., Yodh E., Vekar S., Zackai E.H., Gur R.C., Emanuel B.S., Gur R.E.**	Incidental radiologic findings in the 22q11.2 deletion syndrome	2014	American Journal of Neuroradiology	35	2186	2191	T&A	Excluded	wrong outcome
**Eliez S., Barnea-Goraly N., Schmitt J.E., Liu Y., Reiss A.L.**	Increased basal ganglia volumes in velo-cardio-facial syndrome (deletion 22q11.2)	2002	Biological Psychiatry	52	68	70	T&A	Excluded	wrong outcome
**Shashi V., Francis A., Hooper S.R., Kranz P.G., Zapadka M., Schoch K., Ip E., Tandon N., Howard T.D., Keshavan M.S.**	Increased corpus callosum volume in children with chromosome 22q11.2 deletion syndrome is associated with neurocognitive deficits and genetic polymorphisms	2012	European Journal of Human Genetics	20	1051	1057	T&A	Excluded	wrong outcome
**Beaton E.A., Qin Y., Nguyen V., Johnson J., Pinter J.D., Simon T.J.**	Increased incidence and size of cavum septum pellucidum in children with chromosome 22q11.2 deletion syndrome	2010	Psychiatry Research - Neuroimaging	181	108	113	T&A	Excluded	wrong outcome
**Giersch A., Glaser B., Pasca C., Chabloz M., Debbané M., Eliez S.**	Individuals with 22q11.2 deletion syndrome are impaired at explicit, but not implicit, discrimination of local forms embedded in global structures	2014	American Journal on Intellectual and Developmental Disabilities	119	261	275	T&A	Excluded	wrong outcome
**Samanta D.**	Infantile spasms in Williams–Beuren syndrome with typical deletions of the 7q11.23 critical region and a review of the literature	2017	Acta Neurologica Belgica	117	359	362	T&A	Excluded	wrong topic
**Hantash F.M., Wang B.T., Owen R., Ross L.P., Mahon L.W., Boyar F.Z., Anguiano A., Strom C.M.**	Inherited and de novo 22q11.2 distal duplications in two patients with autistic features, speech delay and no dysmorphology	2012	Journal of Pediatric Genetics	1	115	124	T&A	Excluded	wrong topic
**Cox K., Price V., Kahr W.H.**	Inherited platelet disorders: A clinical approach to diagnosis and management	2011	Expert Review of Hematology	4	455	472	T&A	Excluded	wrong topic
**Nurden A.T., Nurden P.**	Inherited thrombocytopenias	2007	Haematologica	92	1158	1164	T&A	Excluded	wrong topic

**Walter E., Mazaika P.K., Reiss A.L.**	Insights into brain development from neurogenetic syndromes: Evidence from fragile X syndrome, Williams syndrome, Turner syndrome and velocardiofacial syndrome	2009	Neuroscience	164	257	271	T&A	Excluded	wrong study design
**De Smedt B., Devriendt K., Fryns J.-P., Vogels A., Gewillig M., Swillen A.**	Intellectual abilities in a large sample of children with Velo-Cardio-Facial Syndrome: An update	2007	Journal of Intellectual Disability Research	51	666	670	T&A	Excluded	wrong outcome
**Cirillo E., Giardino G., Gallo V., Puliafito P., Azzari C., Bacchetta R., Cardinale F., Cicalese M.P., Consolini R., Martino S., Martire B., Molinatto C., Plebani A., Scarano G., Soresina A., Cancrini C., Rossi P., Digilio M.C., Pignata C.**	Intergenerational and intrafamilial phenotypic variability in 22q11.2 Deletion syndrome subjects	2014	BMC Medical Genetics	15			T&A	Excluded	wrong outcome
**Fernández R.M., Sánchez J., García-Díaz L., Peláez-Nora Y., González-Meneses A., Antiñolo G., Borrego S.**	Interstitial 10p deletion derived from a maternal ins(16;10)(q22;p13p15.2): Report of the first familial case of 10p monosomy affecting to two familial members of different generations	2016	American Journal of Medical Genetics, Part A	170	1268	1273	T&A	Excluded	wrong topic
**Yatsenko S.A., Yatsenko A.N., Szigeti K., Craigen W.J., Stankiewicz P., Cheung S.W., Lupski J.R.**	Interstitial deletion of 10p and atrial septal defect in DiGeorge 2 syndrome	2004	Clinical Genetics	66	128	136	T&A	Excluded	wrong study design
**Barnea-Goraly N., Menon V., Krasnow B., Ko A., Reiss A., Eliez S.**	Investigation of white matter structure in velocardiofacial syndrome: A diffusion tensor imaging study	2003	American Journal of Psychiatry	160	1863	1869	T&A	Excluded	wrong outcome
**Reish O., Finkelstein Y., Mesterman R., Nachmani A., Wolach B., Fejgin M., Amiel A.**	Is isolated palatal anomaly an indication to screen for 22q11 region deletion?	2003	Cleft Palate-Craniofacial Journal	40	176	179	T&A	Excluded	wrong outcome

**Funke B., Puech A., Saint-Jore B., Pandita R., Skoultchi A., Morrow B.**	Isolation and characterization of a human gene containing a nuclear localization signal from the critical region for velo-cardio-facial syndrome on 22q11	1998	Genomics	53	146	154	T&A	Excluded	no human
**Locke B.A., Dasu T., Verbsky J.W.**	Laboratory diagnosis of primary immunodeficiencies	2014	Clinical Reviews in Allergy and Immunology	46	154	168	T&A	Excluded	wrong topic
**Cuneo B.F., Langman C.B., Ilbawi M.N., Ramakrishnan V., Cutilletta A., Driscoll D.A.**	Latent hypoparathyroidism in children with conotruncal cardiac defects	1996	Circulation	93	1702	1708	T&A	Excluded	wrong outcome
**Erdödi L., Lajiness-O'Neill R., Schmitt T.A.**	Learning curve analyses in neurodevelopmental disorders: Are children with autism spectrum disorder truly visual learners?	2013	Journal of Autism and Developmental Disorders	43	880	890	T&A	Excluded	wrong topic
**Scariati E., Padula M.C., Schaer M., Eliez S.**	Long-range dysconnectivity in frontal and midline structures is associated to psychosis in 22q11.2 deletion syndrome	2016	Journal of Neural Transmission	123	823	839	T&A	Excluded	wrong study design
**Radoeva P.D., Bansal R., Antshel K.M., Fremont W., Peterson B.S., Kates W.R.**	Longitudinal study of cerebral surface morphology in youth with 22q11.2 deletion syndrome, and association with positive symptoms of psychosis	2017	Journal of Child Psychology and Psychiatry and Allied Disciplines	58	305	314	T&A	Excluded	wrong outcome
**Lima K., Abrahamsen T.G., Foelling I., Natvig S., Ryder L.P., Olaussen R.W.**	Low thymic output in the 22q11.2 deletion syndrome measured by CCR9 +CD45RA+ T cell counts and T cell receptor rearrangement excision circles	2010	Clinical and Experimental Immunology	161	98	107	T&A	Excluded	wrong outcome
**Mishra P.K.**	Management strategies for interrupted aortic arch with associated anomalies	2009	European Journal of Cardio-thoracic Surgery	35	569	576	T&A	Excluded	wrong topic
**Samuels I.S., Saitta S.C., Landreth G.E.**	MAP'ing CNS Development and Cognition: An ERKsome Process	2009	Neuron	61	160	167	T&A	Excluded	wrong topic

**Lin A., Ching C.R.K., Vajdi A., Sun D., Jonas R.K., Jalbrzikowski M., Kushan-Wells L., Hansen L.P., Krikorian E., Gutman B., Dokoru D., Helleman G., Thompson P.M., Bearden C.E.**	Mapping 22q11.2 gene dosage effects on brain morphometry	2017	Journal of Neuroscience	37	6183	6199	T&A	Excluded	wrong outcome
**Dennis E.L., Thompson P.M.**	Mapping connectivity in the developing brain	2013	International Journal of Developmental Neuroscience	31	525	542	T&A	Excluded	wrong topic
**Hwang V.J., Maar D., Regan J., Angkustsiri K., Simon T.J., Tassone F.**	Mapping the deletion endpoints in individuals with 22q11.2 Deletion Syndrome by droplet digital PCR	2014	BMC Medical Genetics	15			FT	Included	
**De Smedt B., Swillen A., Devriendt K., Fryns J.P., Verschaffel L., Ghesquière P.**	Mathematical disabilities in children with velo-cardio-facial syndrome	2007	Neuropsychologia	45	885	895	T&A	Excluded	wrong outcome
**De Smedt B., Swillen A., Devriendt K., Fryns J.P., Verschaffel L., Ghesquière P.**	Mathematical disabilities in young primary school children with velo-cardio-facial syndrome	2006	Genetic Counseling	17	259	280	T&A	Excluded	wrong outcome
**Shashi V., Berry M.N., Keshavan M.S.**	Mechanistic approach to understanding psychosis risk in velocardiofacial syndrome	2009	Current Pediatric Reviews	5	89	104	T&A	Excluded	wrong study design
**Tan G.M., Arnone D., McIntosh A.M., Ebmeier K.P.**	Meta-analysis of magnetic resonance imaging studies in chromosome 22q11.2 deletion syndrome (velocardiofacial syndrome)	2009	Schizophrenia Research	115	173	181	T&A	Excluded	wrong outcome
**Kerstjens-Frederikse W.S., Kurahashi H., Driscoll D.A., Budarf M.L., Emanuel B.S., Beatty B., Scheidl T., Siegel-Bartelt J., Henderson K., Cytrynbaum C., Nie G., Teshima I.**	Microdeletion 22q11.2: Clinical data and deletion size	1999	Journal of Medical Genetics	36	721	723	FT	Excluded	incomplete outcome
**Carvill G.L., Mefford H.C.**	Microdeletion syndromes	2013	Current Opinion in Genetics and Development	23	232	239	T&A	Excluded	wrong topic
**Seemanová E.**	Microdeletion syndromes [Mikrodelecní syndromy.]	2002	Casopis Lekaru Ceskych	141	363	370	T&A	Excluded	wrong topic

**Ensenauer R.E., Adeyinka A., Flynn H.C., Michels V.V., Lindor N.M., Dawson D.B., Thorland E.C., Lorentz C.P., Goldstein J.L., McDonald M.T., Smith W.E., Simon-Fayard E., Alexander A.A., Kulharya A.S., Ketterling R.P., Clark R.D., Jalal S.M.**	Microduplication 22q11.2, an Emerging Syndrome: Clinical, Cytogenetic, and Molecular Analysis of Thirteen Patients	2003	American Journal of Human Genetics	73	1027	1040	T&A	Excluded	wrong topic
**Van Campenhout S., Devriendt K., Breckpot J., Frijns J.-P., Peeters H., Van Buggenhout G., Van Esch H., Maes B., Swillen A.**	Microduplication 22q11.2: A description of the clinical, developmental and behavioral characteristics during childhood	2012	Genetic Counseling	23	135	148	T&A	Excluded	wrong topic
**Caballero F., Fresán A., Palacios J.J., Rodríguez-Verdugo S.**	Minor physical anomalies and schizophrenia [Anomalías físicas menores y esquizofrenia]	2007	Salud Mental	30	12	19	T&A	Excluded	wrong topic
**Schulze B.R.B., Tariverdian G., Komposch G., Stellzig A.**	Misclassification risk of patients with bilateral cleft lip and palate and manifestations of median facial dysplasia: A new variant of Del(22q11.2) syndrome?	2001	American Journal of Medical Genetics	99	280	285	T&A	Excluded	wrong study design
**Kirchhoff M., Bisgaard A.-M., Bryndorf T., Gerdes T.**	MLPA analysis for a panel of syndromes with mental retardation reveals imbalances in 5.8% of patients with mental retardation and dysmorphic features, including duplications of the Sotos syndrome and Williams-Beuren syndrome regions	2007	European Journal of Medical Genetics	50	33	42	T&A	Excluded	wrong topic
**Meechan D.W., Maynard T.M., Tucker E.S., Fernandez A., Karpinski B.A., Rothblat L.A., LaMantia A.-S.**	Modeling a model: Mouse genetics, 22q11.2 Deletion Syndrome, and disorders of cortical circuit development	2015	Progress in Neurobiology	130	1	28	T&A	Excluded	no human
**Lindstrand A., Malmgren H., Verri A., Benetti E., Eriksson M., Nordgren A., Anderlid B.-M., Golovleva I., Schoumans J., Blennow E.**	Molecular and clinical characterization of patients with overlapping 10p deletions	2010	American Journal of Medical Genetics, Part A	152	1233	1243	T&A	Excluded	wrong topic

**Matsuoka R., Kimura M., Scambler P.J., Morrow B.E., Imamura S.-I., Minoshima S., Shimizu N., Yamagishi H., Joh-o K., Watanabe S., Oyama K., Saji T., Ando M., Takao A., Momma K.**	Molecular and clinical study of 183 patients with conotruncal anomaly face syndrome	1998	Human Genetics	103	70	80	FT	Excluded	incomplete outcome
**Todorov T., Todorova A., Avdjieva D., Dimova P., Angelova L., Tincheva R., Mitev V.**	Molecular basis of mental retardation in a sample from Bulgaria	2010	Genetic Counseling	21	257	262	T&A	Excluded	wrong topic
**Koolen D.A., Reardon W., Rosser E.M., Lacombe D., Hurst J.A., Law C.J., Bongers E.M.H.F., Van Ravenswaaij-Arts C.M., Leisink M.A.R., Van Kessel A.G., Veltman J.A., De Vries B.B.A.**	Molecular characterisation of patients with subtelomeric 22q abnormalities using chromosome specific array-based comparative genomic hybridisation	2005	European Journal of Human Genetics	13	1019	1024	T&A	Excluded	wrong topic
**Iascone M.R., Vittorini S., Sacchelli M., Spadoni I., Simi P., Giusti S.**	Molecular characterization of 22q11 deletion in a three-generation family with maternal transmission	2002	American Journal of Medical Genetics	108	319	321	T&A	Excluded	wrong study design
**Vittorini S., Sacchelli M., Iascone M.R., Collavoli A., Storti S., Giusti A., Andreani G., Botto N., Biagini A., Clerico A.**	Molecular characterization of chromosome 22 deletions by short tandem repeat polymorphism (STRP) in patients with conotruncal heart defects	2001	Clinical Chemistry and Laboratory Medicine	39	1249	1258	FT	Excluded	incomplete outcome
**Torres L., Rosell J., Bernués M., Govea N., Gorreto L., García Algas F., De la Fuente Sánchez M.A., Heine Súñer D.**	Molecular characterization of deletions and mutations of the 22q11 region in patients with congenital heart diseases [Caracterización molecular de deleciones y mutaciones de la región 22q11 en pacientes con cardiopatías congé nitas]	2004	Investigacion Cardiovascular	7	81	92	T&A	Excluded	wrong outcome
**Mears A.J., Duncan A.M.V., Budarf M.L., Emanuel B.S., Sellinger B., Siegel-Bartelt J., Greenberg C.R., McDermid H.E.**	Molecular characterization of the marker chromosome associated with cat eye syndrome	1994	American Journal of Human Genetics	55	134	142	T&A	Excluded	wrong topic

**Carlson C., Sirotkin H., Pandita R., Goldberg R., McKie J., Wadey R., Patanjali S.R., Weissman S.M., Anyane-Yeboa K., Warburton D., Scambler P., Shprintzen R., Kucherlapati R., Morrow B.E.**	Molecular definition of 22q11 deletions in 151 velo-cardio-facial syndrome patients	1997	American Journal of Human Genetics	61	620	629	FT	Excluded	incomplete outcome
**Stachon A.C., Baskin B., Smith A.C., Shugar A., Cytrynbaum C., Fishman L., Mendoza-Londono R., Klatt R., Teebi A., Ray P.N., Weksberg R.**	Molecular diagnosis of 22q11.2 deletion and duplication by multiplex ligation dependent probe amplification	2007	American Journal of Medical Genetics, Part A	143	2924	2930	FT	Excluded	incomplete outcome
**Moore G.E.**	Molecular Genetic Approaches to the Study of Human Craniofacial Dysmorphologies	1995	International Review of Cytology	158	215	277	T&A	Excluded	wrong topic
**Prescott K., Scambler P.J.**	Molecular genetics of velo-cardio-facial syndrome	2005	Velo-Cardio-Facial Syndrome: A Model for Understanding Microdeletion Disorders		19	46	T&A	Excluded	wrong publication type
**Spengler S., Begemann M., Ortiz Brüchle N., Baudis M., Denecke B., Kroisel P.M., Oehl-Jaschkowitz B., Schulze B., Raabe-Meyer G., Spaich C., Blümel P., Jauch A., Moog U., Zerres K., Eggermann T.**	Molecular karyotyping as a relevant diagnostic tool in children with growth retardation with silver-russell features	2012	Journal of Pediatrics	161	933	9420	T&A	Excluded	wrong topic
**Barnett C.P., Van Bon B.W.M.**	Monogenic and chromosomal causes of isolated speech and language impairment	2015	Journal of Medical Genetics	52	719	729	T&A	Excluded	wrong topic
**Mihailov A., Padula M.C., Scariati E., Schaer M., Schneider M., Eliez S.**	Morphological brain changes associated with negative symptoms in patients with 22q11.2 Deletion Syndrome	2017	Schizophrenia Research	188	52	58	T&A	Excluded	wrong outcome
**Dempsey M.A., Schwartz S., Waggoner D.J.**	Mosaicism del(22)(q11.2q11.2)/dup(22)(q11.2q11.2) in a patient with features of 22q11.2 deletion syndrome	2007	American Journal of Medical Genetics, Part A	143	1082	1086	T&A	Excluded	wrong topic
**Van Der Weyden L., Bradley A.**	Mouse chromosome engineering for modeling human disease	2006	Annual Review of Genomics and Human Genetics	7	247	276	T&A	Excluded	no human

**Geyer M.A., McIlwain K.L., Paylor R.**	Mouse genetic models for prepulse inhibition: An early review	2002	Molecular Psychiatry	7	1039	1053	T&A	Excluded	no human
**Piret S.E., Thakker R.V.**	Mouse models for inherited endocrine and metabolic disorders	2011	Journal of Endocrinology	211	211	230	T&A	Excluded	no human
**O'Hanlon E., Howley S., Prasad S., McGrath J., Leemans A., McDonald C., Garavan H., Murphy K.C.**	Multimodal MRI reveals structural connectivity differences in 22q11 deletion syndrome related to impaired spatial working memory	2016	Human Brain Mapping	37	4689	4705	T&A	Excluded	wrong outcome
**Dittwald P., Gambin T., Szafranski P., Li J., Amato S., Divon M.Y., Rojas -L.X.R., Elton L.E., Scott D.A., Schaaf C.P., Torres-Martinez W., Stevens A.K., Rosenfeld J.A., Agadi S., Francis D., Kang S.-H.L., Breman A., Lalani S.R., Bacino C.A., Bi W., Milosavljevic A., Beaudet A.L., Patel A., Shaw C.A., Lupski J.R., Gambin A., Cheung S.W., Stankiewicz P.**	NAHR-mediated copy-number variants in a clinical population: Mechanistic insights into both genomic disorders and Mendelizing traits	2013	Genome Research	23	1395	1409	T&A	Excluded	wrong topic
**Schneider M., Van der Linden M., Menghetti S., Debbané M., Eliez S.**	Negative and paranoid symptoms are associated with negative performance beliefs and social cognition in 22q11.2 deletion syndrome	2017	Early Intervention in Psychiatry	11	156	164	T&A	Excluded	wrong outcome
**Gul K.A., Øverland T., Osnes L., Baumbusch L.O., Pettersen R.D., Lima K., Abrahamsen T.G.**	Neonatal Levels of T-cell Receptor Excision Circles (TREC) in Patients with 22q11.2 Deletion Syndrome and Later Disease Features	2015	Journal of Clinical Immunology	35	408	415	T&A	Excluded	wrong outcome

Demaerel W., Hestand M.S., Vergaelen E., Swillen A., López-Sánchez M., Pérez-Jurado L.A., McDonald-McGinn D.M., Zackai E., Emanuel B.S., Morrow B.E., Breckpot J., Devriendt K., Vermeesch J.R., Antshel K., Arango C., Armando M., Bassett A., Bearden C., Boot E., Bravo-Sanchez M., Breetvelt E., Busa T., Butcher N., Campbell L., Carmel M., Chow E., Crowley T.B., Cubells J., Cutler D., Demaerel W., Digilio M.C., Duijff S., Eliez S., Emanuel B., Epstein M., Evers R., Fernandez Garcia-Moya L., Fiksinski A., Fraguas D., Fremont W., Fritsch R., Garcia-Minaur S., Golden A., Gothelf D., Guo T., Gur R., Gur R., Heine-Suner D., Hestand M., Hooper S., Kates W., Kushan L., Laorden-Nieto A., Maeder J., Marino B., Marshall C., McCabe K., McDonald-McGinn D., Michaelovosky E., Morrow B., Moss E., Mulle J., Murphy D., Murphy K., Murphy C., Niarchou M., Ornstein C., Owen M., Philip N., Repetto G., Schneider M., Shashi V., Simon T., Swillen A., Tassone F., Unolt M., van Amelsvoort T., van den Bree M., Van Duin E., Vergaelen E., Vermeesch J., Vicari S., Vingerhoets C., Vorstman J., Warren S., Weinberger R., Weisman O., Weizman A., Zackai E., Zhang Z., Zwick [illegible]	Nested Inversion Polymorphisms Predispose Chromosome 22q11.2 to Meiotic Rearrangements	2017	American Journal of Human Genetics	101	616	622	T&A	Excluded	wrong outcome
Bearden C.E., Glahn D.C., Lee A.D., Chiang M.-C., van Erp T.G.M., Cannon T.D., Reiss A.L., Toga A.W., Thompson P.M.	Neural phenotypes of common and rare genetic variants	2008	Biological Psychology	79	43	57	T&A	Excluded	wrong topic
Ellegood J., Markx S., Lerch J.P., Steadman P.E., Genç C., Provenzano F., Kushner S.A., Henkelman R.M., Karayiorgou M., Gogos J.A.	Neuroanatomical phenotypes in a mouse model of the 22q11.2 microdeletion	2014	Molecular Psychiatry	19	99	107	T&A	Excluded	no human

**Baker K., Chaddock C.A., Baldeweg T., Skuse D.**	Neuroanatomy in adolescents and young adults with 22q11 Deletion Syndrome: Comparison to an IQ-matched group	2011	NeuroImage	55	491	499	T&A	Excluded	wrong outcome
**Demily C., Rossi M., Schneider M., Edery P., Leleu A., D'Amato T., Franck N., Eliez S.**	Neurocognitive and psychiatric management of the 22q11.2 deletion syndrome [Perspectives actuelles dans la microdélétion 22q11.2: Prise en charge du phénotype neurocomportemental]	2015	Encephale	41	266	273	T&A	Excluded	wrong study design
**Chow E.W.C., Watson M., Young D.A., Bassett A.S.**	Neurocognitive profile in 22q11 deletion syndrome and schizophrenia	2006	Schizophrenia Research	87	270	278	T&A	Excluded	wrong outcome
**Gur R.E.**	Neurodevelopmental genomic strategies in the study of the psychosis spectrum	2016	Nebraska Symposium on Motivation	63	5	30	T&A	Excluded	wrong publication type
**Martinez-Biarge M., Jowett V.C., Cowan F.M., Wusthoff C.J.**	Neurodevelopmental outcome in children with congenital heart disease	2013	Seminars in Fetal and Neonatal Medicine	18	279	285	T&A	Excluded	wrong topic
**Latal B.**	Neurodevelopmental Outcomes of the Child with Congenital Heart Disease	2016	Clinics in Perinatology	43	173	185	T&A	Excluded	wrong topic
**Boot E., van Amelsvoort T.A.M.J.**	Neuroimaging correlates of 22q11.2 deletion syndrome: Implications for schizophrenia research	2012	Current Topics in Medicinal Chemistry	12	2303	2313	T&A	Excluded	wrong study design
**Muraki K., Tanigaki K.**	Neuronal migration abnormalities and its possible implications for schizophrenia	2015	Frontiers in Neuroscience	9			T&A	Excluded	wrong topic
**Kiehl T.R., Chow E.W.C., Mikulis D.J., George S.R., Bassett A.S.**	Neuropathologic features in adults with 22q11.2 deletion syndrome	2009	Cerebral Cortex	19	153	164	T&A	Excluded	wrong outcome
**Zinkstok J., Van Amelsvoort T.**	Neuropsychological profile and neuroimaging in patients with 22q11.2 Deletion Syndrome: A review	2005	Child Neuropsychology	11	21	37	T&A	Excluded	wrong study design

**Śmigiel R., Piotrowicz M., Łaczmańska I., Makowska I., Błońska A., Hoffmann K., Jakubowski L., Blin N., Sasiadek M.M.**	New bacterial artificial chromosome and commercial FISH probes for the 22q11.2 region in patients with congenital heart defect and with phenotype resembling DiGeorge and velocardiofacial syndromes	2007	Advances in Clinical and Experimental Medicine	16	717	723	T&A	Excluded	not available
**Wenger S.L., Boone L.Y., Cummins J.H., Del Vecchio M.A., Bay C.A., Hummel M., Mowery-Rushton P.A.**	Newborn infant with inherited ring and de novo interstitial deletion on homologous chromosome 22s	2000	American Journal of Medical Genetics	91	351	354	T&A	Excluded	wrong topic
**Guipponi M., Santoni F., Schneider M., Gehrig C., Bustillo X.B., Kates W.R., Morrow B., Armando M., Vicari S., Sloan-Béna F., Gagnebin M., Shashi V., Hooper S.R., Eliez S., Antonarakis S.E.**	No evidence for the presence of genetic variants predisposing to psychotic disorders on the non-deleted 22q11.2 allele of VCFS patients	2017	Translational Psychiatry	7			T&A	Excluded	wrong outcome
**Lepach A.C., Petermann F.**	Nonverbal and verbal learning: A comparative study of children and adolescents with 22q11 deletion syndrome, non-syndromal Nonverbal Learning Disorder and memory disorder	2011	Neurocase	17	480	490	T&A	Excluded	wrong outcome
**Torti E.E., Braddock S.R., Bernreuter K., Batanian J.R.**	Oculo-auriculo-vertebral spectrum, cat eye, and distal 22q11 microdeletion syndromes: A unique double rearrangement	2013	American Journal of Medical Genetics, Part A	161	1992	1998	T&A	Excluded	wrong study design
**Sikora A.G., Lee K.C.**	Otolaryngologic manifestations of immunodeficiency	2003	Otolaryngologic Clinics of North America	36	647	672	T&A	Excluded	wrong topic
**Moutin E., Nikonenko I., Stefanelli T., Wirth A., Ponimaskin E., De Roo M., Muller D.**	Palmitoylation of cdc42 Promotes Spine Stabilization and Rescues Spine Density Deficit in a Mouse Model of 22q11.2 Deletion Syndrome	2017	Cerebral Cortex	27	3618	3629	T&A	Excluded	no human

**Eliez S., Antonarakis S.E., Morris M.A., Dahoun S.P., Reiss A.L.**	Parental origin of the deletion 22q11.2 and brain development in velocardiofacial syndrome: A preliminary study	2001	Archives of General Psychiatry	58	64	68	T&A	Excluded	wrong outcome
**Van Den Heuvel E., Manders E., Swillen A., Zink I.**	Parental report on socio-communicative behaviours in children with 22q11.2 deletion syndrome	2017	Journal of Intellectual and Developmental Disability	42	162	172	T&A	Excluded	wrong outcome
**León L.E., Benavides F., Espinoza K., Vial C., Alvarez P., Palomares M., Lay-Son G., Miranda M., Repetto G.M.**	Partial microduplication in the histone acetyltransferase complex member KANSL1 is associated with congenital heart defects in 22q11.2 microdeletion syndrome patients	2017	Scientific Reports	7			T&A	Excluded	wrong outcome
**Yi J.J., Weinberger R., Moore T.M., Calkins M.E., Guri Y., McDonald-McGinn D.M., Zackai E.H., Emanuel B.S., Gur R.E., Gothelf D., Gur R.C.**	Performance on a computerized neurocognitive battery in 22q11.2 deletion syndrome: A comparison between US and Israeli cohorts	2016	Brain and Cognition	106	33	41	T&A	Excluded	wrong outcome
**Schell D.N., Winlaw D.S.**	Peri-operative management of paediatric patients undergoing cardiac surgery - focus on respiratory aspects of care	2007	Paediatric Respiratory Reviews	8	336	347	T&A	Excluded	wrong topic
**Van Kogelenberg M., Ghedia S., McGillivray G., Bruno D., Leventer R., MacDermot K., Nelson J., Nagarajan L., Veltman J.A., De Brouwer A.P., McKinlay Gardner R.J., Van Bokhoven H., Kirk E.P., Robertson S.**	Periventricular heterotopia in common microdeletion syndromes	2010	Molecular Syndromology	1	35	41	T&A	Excluded	wrong topic
**Weischenfeldt J., Symmons O., Spitz F., Korbel J.O.**	Phenotypic impact of genomic structural variation: Insights from and for human disease	2013	Nature Reviews Genetics	14	125	138	T&A	Excluded	wrong topic
**Cancrini C., Romiti M.L., Finocchi A., Di Cesare S., Ciaffi P., Capponi C., Pahwa S., Rossi P.**	Post-natal ontogenesis of the T-cell receptor CD4 and CD8 Vβ repertoire and immune function in children with DiGeorge syndrome	2005	Journal of Clinical Immunology	25	265	274	T&A	Excluded	wrong outcome

**Galindo A., Gutiérrez-Larraya F., Martínez J.M., Del Rio M., Grañeras A., Velasco J.M., Puerto B., Gratacos E.**	Prenatal diagnosis and outcome for fetuses with congenital absence of the pulmonary valve	2006	Ultrasound in Obstetrics and Gynecology	28	32	39	T&A	Excluded	wrong topic
**Razavi R.S., Sharland G.K., Simpson J.M.**	Prenatal diagnosis by echocardiogram and outcome of absent pulmonary valve syndrome	2003	American Journal of Cardiology	91	429	432	T&A	Excluded	wrong topic
**Driscoll D.A.**	Prenatal diagnosis of the 22q11.2 deletion syndrome	2001	Genetics in Medicine	3	14	18	T&A	Excluded	wrong study design
**Hunter L.E., Simpson J.M.**	Prenatal screening for structural congenital heart disease	2014	Nature Reviews Cardiology	11	323	334	T&A	Excluded	wrong topic
**Bonilla F.A.**	Presenting phenotype in 100 children with the 22q11 deletion syndrome: Commentary	2006	Pediatrics	118	S47	S48	FT	Excluded	incomplete outcome
**Hoogendoorn M.L.C., Vorstman J.A.S., Jalali G.R., Selten J.-P., Sinke R.J., Emanuel B.S., Kahn R.S.**	Prevalence of 22q11.2 deletions in 311 Dutch patients with schizophrenia	2008	Schizophrenia Research	98	84	88	T&A	Excluded	wrong outcome
**Zhang Y., Wang Z., Gemeinhart R.A.**	Progress in microRNA delivery	2013	Journal of Controlled Release	172	962	974	T&A	Excluded	wrong topic
**Favier R., Raslova H.**	Progress in understanding the diagnosis and molecular genetics of macrothrombocytopenias	2015	British Journal of Haematology	170	626	639	T&A	Excluded	wrong topic
**Sugama S., Namihira T., Matsuoka R., Taira N., Eto Y., Maekawa K.**	Psychiatric inpatients and chromosome deletions within 22q11.2	1999	Journal of Neurology Neurosurgery and Psychiatry	67	803	806	T&A	Excluded	wrong outcome
**Jolin E.M., Weller R.A., Weller E.B.**	Psychosis in children with velocardiofacial syndrome (22q11.2 deletion syndrome)	2009	Current Psychiatry Reports	11	99	105	T&A	Excluded	wrong outcome
**Devriendt K., Swillen A., Stalmans I., Casteels I.**	Pulmonary atresia/ventricular septal defect associated with facial port-wine stain and retinal vascular abnormality: A new constellation or deletion in chromosome 22q11.2? [7]	2005	American Journal of Medical Genetics	132 A	340	341	T&A	Excluded	wrong topic

**Chow E.W.C., Mikulis D.J., Zipursky R.B., Scutt L.E., Weksberg R., Bassett A.S.**	Qualitative MRI findings in adults with 22q11 deletion syndrome and schizophrenia	1999	Biological Psychiatry	46	1436	1442	T&A	Excluded	wrong outcome
**Padula M.C., Schaer M., Scariati E., Mutlu A.K., Zöller D., Schneider M., Eliez S.**	Quantifying indices of short- and long-range white matter connectivity at each cortical vertex	2017	PLoS ONE	12	1436	1442	T&A	Excluded	wrong topic
**Paronett E.M., Meechan D.W., Karpinski B.A., LaMantia A.-S., Maynard T.M.**	Ranbp1, deleted in DiGeorge/22q11.2 deletion syndrome, is a microcephaly gene that selectively disrupts layer 2/3 cortical projection neuron generation	2015	Cerebral Cortex	25	3977	3993	T&A	Excluded	wrong topic
**Mlynarski E.E., Xie M., Taylor D., Sheridan M.B., Guo T., Racedo S.E., McDonald-McGinn D.M., Chow E.W.C., Vorstman J., Swillen A., Devriendt K., Breckpot J., Digilio M.C., Marino B., Dallapiccola B., Philip N., Simon T.J., Roberts A.E., Piotrowicz M., Bearden C.E., Eliez S., Gothelf D., Coleman K., Kates W.R., Devoto M., Zackai E., Heine-Suñer D., Goldmuntz E., Bassett A.S., Morrow B.E., Emanuel B.S.**	Rare copy number variants and congenital heart defects in the 22q11.2 deletion syndrome	2016	Human Genetics	135	273	285	FT	Excluded	incomplete outcome
**Fulcoli F.G., Franzese M., Liu X., Zhang Z., Angelini C., Baldini A.**	Rebalancing gene haploinsufficiency in vivo by targeting chromatin	2016	Nature Communications	7	273	285	T&A	Excluded	no human
**Dauvé V., McLin V.A.**	Recent advances in the molecular and genetic understanding of congenital gastrointestinal malformations	2013	Journal of Pediatric Gastroenterology and Nutrition	57	4	13	T&A	Excluded	wrong topic

**De Kovel C.G.F., Trucks H., Helbig I., Mefford H.C., Baker C., Leu C., Kluck C., Muhle H., Von Spiczak S., Ostertag P., Obermeier T., Kleefuß-Lie A.A., Hallmann K., Steffens M., Gaus V., Klein K.M., Hamer H.M., Rosenow F., Brilstra E.H., Kasteleijn-Nolst Trenité D., Swinkels M.E.M., Weber Y.G., Unterberger I., Zimprich F., Urak L., Feucht M., Fuchs K., Møller R.S., Hjalgrim H., De Jonghe P., Suls A., Rückert I.-M., Wichmann H.-E., Franke A., Schreiber S., Nürnberg P., Elger C.E., Lerche H., Stephani U., Koeleman B.P.C., Lindhout D., Eichler E.E., Sander**	Recurrent microdeletions at 15q11.2 and 16p13.11 predispose to idiopathic generalized epilepsies	2010	Brain	133	23	32	T&A	Excluded	wrong topic
**Bittel D.C., Yu S., Newkirk H., Kibiryeva N., Holt Iii A., Butler M.G., Cooley L.D.**	Refining the 22q11.2 deletion breakpoints in DiGeorge syndrome by aCGH	2009	Cytogenetic and Genome Research	124	113	120	FT	Excluded	incomplete outcome
**Bearden C.E., van Erp T.G.M., Monterosso J.R., Simon T.J., Glahn D.C., Saleh P.A., Hill N.M., McDonald-McGinn D.M., Zackai E., Emanuel B.S., Cannon T.D.**	Regional brain abnormalities in 22q11.2 deletion syndrome: Association with cognitive abilities and behavioral symptoms	2004	Neurocase	10	198	206	T&A	Excluded	wrong outcome
**Schaer M., Glaser B., Ottet M.-C., Schneider M., Cuadra M.B., Debbané M., Thiran J.-P., Eliez S.**	Regional cortical volumes and congenital heart disease: A MRI study in 22q11.2 deletion syndrome	2010	Journal of Neurodevelopmental Disorders	2	224	234	T&A	Excluded	wrong outcome
**Kates W.R., Burnette C.P., Jabs E.W., Rutberg J., Murphy A.M., Grados M., Geraghty M., Kaufmann W.E., Pearlson G.D.**	Regional cortical white matter reductions in velocardiofacial syndrome: A volumetric MRI analysis	2001	Biological Psychiatry	49	677	684	T&A	Excluded	wrong outcome
**Yoshida K., Kuo F., George E.L., Sharpe A.H., Dutta A.**	Requirement of CDC45 for postimplantation mouse development	2001	Molecular and Cellular Biology	21	4598	4603	T&A	Excluded	no human

**Varga I., Plank L., Meštanová V., Zábojníková L.**	Review and proposal for classification of the congenital anomalies of thymus in children [Prehlad a návrh klasifikácie vrodených anomálií týmusu u detí]	2014	Cesko-Slovenska Pediatrie	69	178	190	T&A	Excluded	wrong topic
**Inman K.E., Ezin M., Bronner-Fraser M., Trainor P.A.**	Role of Cardiac Neural Crest Cells in Morphogenesis of the Heart and Great Vessels	2010	Heart Development and Regeneration	69	417	439	T&A	Excluded	wrong publication type
**Mohanty V., Gökmen-Polar Y., Badve S., Janga S.C.**	Role of lncRNAs in health and disease-size and shape matter	2015	Briefings in Functional Genomics	14	115	129	T&A	Excluded	wrong topic
**Tian J., An X., Niu L.**	Role of microRNAs in cardiac development and disease (Review)	2017	Experimental and Therapeutic Medicine	13	3	8	T&A	Excluded	wrong topic
**Bassett A.S., Chow E.W.C.**	Schizophrenia and 22q11.2 deletion syndrome	2008	Current Psychiatry Reports	10	148	157	T&A	Excluded	wrong study design
**Murphy K.C.**	Schizophrenia and velo-cardio-facial syndrome	2002	Lancet	359	426	430	T&A	Excluded	wrong study design
**Karayiorgou M., Morris M.A., Morrow B., Shprintzen R.J., Goldberg R., Borrow J., Gos A., Nestadt G., Wolyniec P.S., Lasseter V.K., Eisen H., Childs B., Kazazizan H.H., Kucherlapati R., Antonarakis S.E., Pulver A.E., Housman D.E.**	Schizophrenia susceptibility associated with interstitial deletions of chromosome 22q11	1995	Proceedings of the National Academy of Sciences of the United States of America	92	7612	7616	T&A	Excluded	wrong outcome
**Williams H.J., Monks S., Murphy K.C., Kirov G., O'Donovan M.C., Owen M.J.**	Schizophrenia two-hit hypothesis in velo-cardio facial syndrome	2013	American Journal of Medical Genetics, Part B: Neuropsychiatric Genetics	162	177	182	T&A	Excluded	wrong outcome
**Pretto D., Maar D., Yrigollen C.M., Regan J., Tassone F.**	Screening newborn blood spots for 22q11.2 deletion syndrome using multiplex droplet digital PCR	2015	Clinical Chemistry	61	182	190	T&A	Excluded	wrong outcome
**Jurewicz I., Owen R.J., O'Donovan M.C., Owen M.J.**	Searching for susceptibility genes in schizophrenia	2001	European Neuropsychopharmacology	11	395	398	T&A	Excluded	wrong topic

**Zemble R., Luning Prak E., McDonald K., McDonald-McGinn D., Zackai E., Sullivan K.**	Secondary immunologic consequences in chromosome 22q11.2 deletion syndrome (DiGeorge syndrome/velocardiofacial syndrome)	2010	Clinical Immunology	136	409	418	T&A	Excluded	wrong outcome
**Antshel K.M., Abdulsabur N., Roizen N., Fremont W., Kates W.R.**	Sex differences in cognitive functioning in velocardiofacial syndrome (VCFS)	2005	Developmental Neuropsychology	28	849	869	T&A	Excluded	wrong outcome
**Wang Q., Charisi A., Latecki L.J., Gee J., Megalooikonomou V.**	Shape similarity analysis of regions of interest in medical images	2010	Progress in Biomedical Optics and Imaging - Proceedings of SPIE	7624	849	869	T&A	Excluded	wrong topic
**Heike C.L., Starr J.R., Rieder M.J., Cunningham M.L., Edwards K.L., Stanaway I.B., Crawford D.C.**	Single nucleotide polymorphism discovery in TBX1 in individuals with and without 22q11.2 deletion syndrome	2010	Birth Defects Research Part A - Clinical and Molecular Teratology	88	54	63	T&A	Excluded	wrong outcome
**PIerpont J.W., Erickson R.P., Thompson F.H., Yang J.-M.**	Size of 22q deletions in four previously reported patients with conotruncal anomaly face syndrome	1996	Clinical Genetics	50	545	547	FT	Excluded	incomplete outcome
**Heike C.L., Avellino A.M., Mirza S.K., Kifle Y., Perkins J., Sze R., Egbert M., Hing A.V.**	Sleep disturbances in 22q11.2 deletion syndrome: A case with obstructive and central sleep apnea	2007	Cleft Palate-Craniofacial Journal	44	340	346	T&A	Excluded	wrong study design
**Lüerßen K., Pruggmayer M., Ptok M.**	Small deletion - Large effect [Kleine Deletion - Große Auswirkung]	2004	HNO	52	258	260	T&A	Excluded	wrong study design
**Halder A., Jain M., Kalsi A.K.**	SNP Microarray in FISH Negative Clinically Suspected 22q11.2 Microdeletion Syndrome	2016	Scientifica	2016			T&A	Excluded	wrong outcome
**Niccols A., Thomas K., Schmidt L.A.**	Socioemotional and Brain Development in Children with Genetic Syndromes Associated with Developmental Delay	2012	The Oxford Handbook of Intellectual Disability and Development				T&A	Excluded	wrong publication type

**Bish J.P., Pendyal A., Ding L., Ferrante H., Nguyen V., McDonald-McGinn D., Zackai E., Simon T.J.**	Specific cerebellar reductions in children with chromosome 22q11.2 deletion syndrome	2006	Neuroscience Letters	399	245	248	T&A	Excluded	wrong outcome
**Rakonjac M., Cuturilo G., Stevanovic M., Jovanovic I., Dobrijevic L.J., Mijovic M., Drakulic D.**	Speech and language abilities of children with the familial form of 22Q11.2 deletion syndrome	2016	Genetika	48	57	72	FT	Excluded	incomplete outcome
**Brandão G.R., De Souza Freitas J.A., Genaro K.F., Yamashita R.P., Fukushiro A.P., Lauris J.R.P.**	Speech outcomes and velopharyngeal function after surgical treatment of velopharyngeal insufficiency in individuals with signs of velocardiofacial syndrome	2011	Journal of Craniofacial Surgery	22	1736	1742	T&A	Excluded	wrong outcome
**Jalbrzikowski M., Jonas R., Senturk D., Patel A., Chow C., Green M.F., Bearden C.E.**	Structural abnormalities in cortical volume, thickness, and surface area in 22q11.2 microdeletion syndrome: Relationship with psychotic symptoms	2013	NeuroImage: Clinical	3	405	415	T&A	Excluded	wrong outcome
**Baylis A.L., Watson P.J., Moller K.T.**	Structural and functional causes of hypernasality in velocardiofacial syndrome	2009	Folia Phoniatrica et Logopaedica	61	93	96	T&A	Excluded	wrong outcome
**Van Amelsvoort T., Daly E., Robertson D., Suckling J., Ng V., Critchley H., Owen M.J., Henry J., Murphy K.C., Murphy D.G.M.**	Structural brain abnormalities associated with deletion at chromosome 22q11: Quantitative neuroimaging study of adults with velo-cardio-facial syndrome	2001	British Journal of Psychiatry	178	412	419	T&A	Excluded	wrong outcome
**Chow E.W.C., Zipursky R.B., Mikulis D.J., Bassett A.S.**	Structural brain abnormalities in patients with schizophrenia and 22q11 Deletion Syndrome	2002	Biological Psychiatry	51	208	215	T&A	Excluded	wrong outcome
**Glaser B., Schaer M., Berney S., Debbane M., Vuilleumier P., Eliez S.**	Structural changes to the fusiform gyrus: A cerebral marker for social impairments in 22q11.2 deletion syndrome?	2007	Schizophrenia Research	96	82	86	T&A	Excluded	wrong outcome

**Lorain S., Demczuk S., Lamour V., Toth S., Aurias A., Roe B.A., Lipinski M.**	Structural organization of the WD repeat protein-encoding gene HIRA in the DiGeorge syndrome critical region of human chromosome 22	1996	Genome Research	6	43	50	T&A	Excluded	no human
**Novelli G., Mari A., Amati F., Colosimo A., Sangiuolo F., Bengala M., Conti E., Ratti A., Bordoni R., Pizzuti A., Baldini A., Crinelli R., Pandolfi F., Magnani M., Dallapiccola B.**	Structure and expression of the human ubiquitin fusion - Degradation gene (UFD1L)	1998	Biochimica et Biophysica Acta - Gene Structure and Expression	1396	158	162	T&A	Excluded	wrong topic
**Yushkevich P.A., Zhang H., Simon T.J., Gee J.C.**	Structure-specific statistical mapping of white matter tracts	2009	Mathematics and Visualization		83	112	T&A	Excluded	wrong topic
**Lindsay E.A., Greenberg F., Shaffer L.G., Shapira S.K., Scambler P.J., Baldini A.**	Submicroscopic deletions at 22q11.2: Variability of the clinical picture and delineation of a commonly deleted region	1995	American Journal of Medical Genetics	56	191	197	FT	Excluded	incomplete outcome
**Sinderberry B., Brown S., Hammond P., Stevens A.F., Schall U., Murphy D.G.M., Murphy K.C., Campbell L.E.**	Subtypes in 22q11.2 deletion syndrome associated with behaviour and neurofacial morphology	2013	Research in Developmental Disabilities	34	116	125	T&A	Excluded	wrong outcome
**Milczuk H.A., Smith D.S., Brockman J.H.**	Surgical outcomes for velopharyngeal insufficiency in velocardiofacial syndrome and nonsyndromic patients	2007	Cleft Palate-Craniofacial Journal	44	412	417	T&A	Excluded	wrong outcome
**Crabtree G., Gogos J.A.**	Synaptic plasticity, neural circuits and the emerging role of altered short-term information processing in schizophrenia	2014	Frontiers in Synaptic Neuroscience	6	412	417	T&A	Excluded	wrong topic
**Habel A., Mcginn M.-J., Zackai E.H., Unanue N., Mcdonald-Mcginn D.M.**	Syndrome-specific growth charts for 22q11.2 deletion syndrome in Caucasian children	2012	American Journal of Medical Genetics, Part A	158 A	2665	2671	T&A	Excluded	wrong outcome
**Pierdominici M., Marziali M., Giovannetti A., Oliva A., Rosso R., Marino B., Digilio M.C., Giannotti A., Novelli G., Dallapiccola B., Aiuti F., Pandolfi F.**	T cell receptor repertoire and function in patients with DiGeorge syndrome and velocardiofacial syndrome	2000	Clinical and Experimental Immunology	121	127	132	T&A	Excluded	wrong outcome

**Douglas N.C., Washkowitz A.J., Naiche L.A., Papaioannou V.E.**	T-Box Genes and Developmental Anomalies	2014	Principles of Developmental Genetics: Second Edition		635	652	T&A	Excluded	wrong topic
**Piliero L.M., Sanford A.N., McDonald-McGinn D.M., Zackai E.H., Sullivan K.E.**	T-cell homeostasis in humans with thymic hypoplasia due to chromosome 22q11.2 deletion syndrome	2004	Blood	103	1020	1025	T&A	Excluded	wrong outcome
**Cao H., Florez S., Amen M., Huynh T., Skobe Z., Baldini A., Amendt B.A.**	Tbx1 regulates progenitor cell proliferation in the dental epithelium by modulating Pitx2 activation of p21	2010	Developmental Biology	347	289	300	T&A	Excluded	no human
**Dastjerdi A., Robson L., Walker R., Hadley J., Zhang Z., Rodriguez-Niedenführ M., Ataliotis P., Baldini A., Scambler P., Francis-West P.**	Tbx1 regulation of myogenic differentiation in the limb and cranial mesoderm	2007	Developmental Dynamics	236	353	363	T&A	Excluded	no human
**Kates W.R., Miller A.M., AbdulSabur N., Antshel K.M., Conchelos J., Fremont W., Roizen N.**	Temporal lobe anatomy and psychiatric symptoms in velocardiofacial syndrome (22q11.2 deletion syndrome)	2006	Journal of the American Academy of Child and Adolescent Psychiatry	45	587	595	T&A	Excluded	wrong outcome
**Bish J.P., Nguyen V., Ding L., Ferrante S., Simon T.J.**	Thalamic reductions in children with chromosome 22q11.2 deletion syndrome	2004	NeuroReport	15	1413	1415	T&A	Excluded	wrong outcome
**Fagman H., Liao J., Westerlund J., Andersson L., Morrow B.E., Nilsson M.**	The 22q11 deletion syndrome candidate gene Tbx1 determines thyroid size and positioning	2007	Human Molecular Genetics	16	276	285	T&A	Excluded	no human
**Papangeli I., Scambler P.**	The 22q11 deletion: DiGeorge and velocardiofacial syndromes and the role of TBX1	2013	Wiley Interdisciplinary Reviews: Developmental Biology	2	393	403	T&A	Excluded	wrong study design
**Richard A.C., Rovelet-Lecrux A., Delaby E., Charbonnier C., Thiruvahindrapuram B., Hatchwell E., Eis P.S., Afenjar A., Dussardier B.G., Scherer S.W., Betancur C., Campion D.**	The 22q11 PRODH/DGCR6 deletion is frequent in hyperprolinemic subjects but is not a strong risk factor for ASD	2016	American Journal of Medical Genetics, Part B: Neuropsychiatric Genetics	171	377	382	T&A	Excluded	wrong outcome

**Jonas R.K., Montojo C.A., Bearden C.E.**	The 22q11.2 deletion syndrome as a window into complex neuropsychiatric disorders over the lifespan	2014	Biological Psychiatry	75	351	360	T&A	Excluded	wrong study design
**Lambert M.P., Arulselvan A., Schott A., Markham S.J., Crowley T.B., Zackai E.H., Mcdonald-Mcginn D.M.**	The 22q11.2 deletion syndrome: Cancer predisposition, platelet abnormalities and cytopenias	2017	American Journal of Medical Genetics, Part A				T&A	Excluded	wrong study design
**Drew L.J., Crabtree G.W., Markx S., Stark K.L., Chaverneff F., Xu B., Mukai J., Fenelon K., Hsu P.-K., Gogos J.A., Karayiorgou M.**	The 22q11.2 microdeletion: Fifteen years of insights into the genetic and neural complexity of psychiatric disorders	2011	International Journal of Developmental Neuroscience	29	259	281	T&A	Excluded	wrong study design
**Van Der Bom T., Zomer A.C., Zwinderman A.H., Meijboom F.J., Bouma B.J., Mulder B.J.M.**	The changing epidemiology of congenital heart disease	2011	Nature Reviews Cardiology	8	50	60	T&A	Excluded	wrong topic
**Gothelf B., Furfaro J.A., Penniman L.C., Glover G.H., Reiss A.L.**	The contribution of novel brain imaging techniques to understanding the neurobiology of mental retardation and developmental disabilities	2005	Mental Retardation and Developmental Disabilities Research Reviews	11	331	339	T&A	Excluded	wrong topic
**Fletcher A.L., Calder A., Hince M.N., Boyd R.L., Chidgey A.P.**	The contribution of thymic stromal abnormalities to autoimmune disease	2011	Critical Reviews in Immunology	31	171	187	T&A	Excluded	wrong topic
**Muldoon M., Ousley O.Y., Kobrynski L.J., Patel S., Oster M.E., Fernandez-Carriba S., Cubells J.F., Coleman K., Pearce B.D.**	The effect of hypocalcemia in early childhood on autism-related social and communication skills in patients with 22q11 deletion syndrome	2015	European Archives of Psychiatry and Clinical Neuroscience	265	519	524	T&A	Excluded	wrong outcome
**Crespi B., Badcock C.**	The evolutionary social brain: From genes to psychiatric conditions	2008	Behavioral and Brain Sciences	31	284	296	T&A	Excluded	wrong topic
**Deak K.L., Horn S.R., Rehder C.W.**	The evolving picture of microdeletion/microduplication syndromes in the age of microarray analysis: Variable expressivity and genomic complexity	2011	Clinics in Laboratory Medicine	31	543	564	T&A	Excluded	wrong topic

**Green T., Steingart L., Frisch A., Zarchi O., Weizman A., Gothelf D.**	The feasibility and safety of S-adenosyl-L-methionine (SAMe) for the treatment of neuropsychiatric symptoms in 22q11.2 deletion syndrome: A double-blind placebo-controlled trial	2012	Journal of Neural Transmission	119	1417	1423	T&A	Excluded	wrong outcome
**Scott J.A., Goodrich-Hunsaker N., Kalish K., Lee A., Hunsaker M.R., Schumann C.M., Carmichael O.T., Simon T.J.**	The hippocampi of children with chromosome 22q11.2 deletion syndrome have localized anterior alterations that predict severity of anxiety	2016	Journal of Psychiatry and Neuroscience	41	203	213	T&A	Excluded	wrong outcome
**Badcock C.**	The imprinted brain: How genes set the balance between autism and psychosis	2011	Epigenomics	3	345	359	T&A	Excluded	wrong topic
**Li-Ling J.**	The Jing-Mai connections of the Heart	2003	International Journal of Cardiology	89	1	11	T&A	Excluded	wrong topic
**Mølsted K., Boers M., Kjär I.**	The morphology of the sella turcica in velocardiofacial syndrome suggests involvement of a neural crest developmental field	2010	American Journal of Medical Genetics, Part A	152	1450	1457	T&A	Excluded	wrong outcome
**Ten Donkelaar H.J., Vermeij-Keers C., Mathijssen I.M.J.**	The neural crest and craniofacial malformations	2014	Clinical Neuroembryology: Development and Developmental Disorders of the Human Central Nervous System		219	269	T&A	Excluded	wrong topic
**Keyte A., Hutson M.R.**	The neural crest in cardiac congenital anomalies	2012	Differentiation	84	25	40	T&A	Excluded	wrong topic
**Mikhail F.M., Burnside R.D., Rush B., Ibrahim J., Godshalk R., Rutledge S.L., Robin N.H., Descartes M.D., Carroll A.J.**	The recurrent distal 22q11.2 microdeletions are often de novo and do not represent a single clinical entity: A proposed categorization system	2014	Genetics in Medicine	16	92	100	T&A	Excluded	wrong topic

**Evers L.J.M., Engelen J.J.M., Houben L.M.H., Curfs L.M.G., van Amelsvoort T.A.M.J.**	The use of two different MLPA kits in 22q11.2 deletion syndrome	2016	European Journal of Medical Genetics	59	183	188	FT	Excluded	incomplete outcome
**Lin A.E.**	The value of aortic valve and aortic arch imaging in the identification of genetic syndromes	1996	Echocardiography	13	447	457	T&A	Excluded	wrong topic
**Vantrappen G., Rommel N., Cremers C.W.R.J., Devriendt K., Frijns J.P.**	The velo-cardio-facial syndrome: The otorhinolaryngeal manifestations and implications	1998	International Journal of Pediatric Otorhinolaryngology	45	133	141	T&A	Excluded	wrong study design
**Veneri D., Franchini M., Randon F., Nichele I., Pizzolo G., Ambrosetti A.**	Thrombocytopenias: A clinical point of view	2009	Blood Transfusion	7	75	85	T&A	Excluded	wrong topic
**Karl K., Heling K.-S., Sarut Lopez A., Thiel G., Chaoui R.**	Thymic-thoracic ratio in fetuses with trisomy 21, 18 or 13	2012	Ultrasound in Obstetrics and Gynecology	40	412	417	T&A	Excluded	wrong topic
**Markert M.L., Sarzotti M., Ozaki D.A., Sempowski G.D., Rhein M.E., Hale L.P., Le Deist F., Alexieff M.J., Li J., Hauser E.R., Haynes B.F., Rice H.E., Skinner M.A., Mahaffey S.M., Jaggers J., Stein L.D., Mill M.R.**	Thymus transplantation in complete DiGeorge syndrome: Immunologic and safety evaluations in 12 patients	2003	Blood	102	1121	1130	T&A	Excluded	wrong outcome
**Stagi S., Lapi E., Gambineri E., Salti R., Genuardi M., Colarusso G., Conti C., Jenuso R., Chiarelli F., Azzari C., De Martino M.**	Thyroid function and morphology in subjects with microdeletion of chromosome 22q11 (del(22)(q11))	2010	Clinical Endocrinology	72	839	844	T&A	Excluded	wrong outcome
**Gabriel Mounir D., Debbané M., Schaer M., Glaser B., Eliez S.**	Time processing in the velo-cardio-facial syndrome (22q11) and its link with the caudate nucleus [Noyau caudé et traitement temporel dans le syndrome vélocardiofacial (22q11)]	2011	Encephale	37	S42	S49	T&A	Excluded	wrong outcome
**Marino B., Digilio M.C., Novelli G., Giannotti A., Dallapiccola B.**	Tricuspid atresia and 22q11 deletion	1997	American Journal of Medical Genetics	72	40	42	T&A	Excluded	wrong outcome
**Dennis E.L., Thompson P.M.**	Typical and atypical brain development: A review of neuroimaging studies	2013	Dialogues in Clinical Neuroscience	15	359	384	T&A	Excluded	wrong topic

**Sandrin-Garcia P., Abramides D.V.M., Martelli L.R., Ramos E.S., Richieri-Costa A., Passos G.A.S.**	Typical phenotypic spectrum of velocardiofacial syndrome occurs independently of deletion size in chromosome 22q11.2	2007	Molecular and Cellular Biochemistry	303	9	17	FT	Excluded	incomplete outcome
**Yang C., Huang C.-H., Cheong M.-L., Hung K.-L., Lin L.-H., Yu Y.-S., Chien C.-C., Huang H.-C., Chen C.-W., Huang C.-J.**	Unambiguous molecular detections with multiple genetic approach for the complicated chromosome 22q11 deletion syndrome	2009	BMC Medical Genetics	10			FT	Excluded	incomplete outcome
**Zrnová E., Vranová V., Šoukalová J., Slámová I., Vilémová M., Gaillyová R., Kuglík P.**	Unique combination of 22q11 and 14qter microdeletion syndromes detected using oligonucleotide array-CGH	2012	Molecular Syndromology	2	88	93	T&A	Excluded	wrong study design
**Jackowski A.P., Laureano M.R., Del'Aquilla M.A., de Moura L.M., Assunção I., Silva I., Schwartzman J.S.**	Update on Clinical Features and Brain Abnormalities in Neurogenetics Syndromes	2011	Journal of Applied Research in Intellectual Disabilities	24	217	236	T&A	Excluded	wrong topic
**Chegar B.E., Tatum III S.A., Marrinan E., Shprintzen R.J.**	Upper airway asymmetry in velo-cardio-facial syndrome	2006	International Journal of Pediatric Otorhinolaryngology	70	1375	1381	T&A	Excluded	wrong outcome
**Bejjani B.A., Saleki R., Ballif B.C., Rorem E.A., Sundin K., Theisen A., Kashork C.D., Shaffer L.G.**	Use of targeted array-based CGH for the clinical diagnosis of chromosomal imbalance: Is less more?	2005	American Journal of Medical Genetics	134 A	259	267	T&A	Excluded	wrong topic
**Solomon B.D.**	VACTERL/VATER association	2011	Orphanet Journal of Rare Diseases	6			T&A	Excluded	wrong topic
**Motzkin B., Marion R., Goldberg R., Shprintzen R., Saenger P.**	Variable phenotypes in velocardiofacial syndrome with chromosomal deletion	1993	The Journal of Pediatrics	123	406	410	FT	Excluded	incomplete outcome
**Guo X., Delio M., Haque N., Castellanos R., Hestand M.S., Vermeesch J.R., Morrow B.E., Zheng D.**	Variant discovery and breakpoint region prediction for studying the human 22q11.2 deletion using BAC clone and whole genome sequencing analysis	2015	Human Molecular Genetics	25	3754	3767	T&A	Excluded	wrong outcome

**Murphy K.C., Owen M.J.**	Velo-cardio-facial syndrome: A model for understanding the genetics and pathogenesis of schizophrenia	2001	British Journal of Psychiatry	179	397	402	T&A	Excluded	wrong study design
**Arnold P.D., Siegel-Bartelt J., Cytrynbaum C., Teshima I., Schachar R.**	Velo-cardio-facial syndrome: Implications of microdeletion 22q11 for schizophrenia and mood disorders	2001	American Journal of Medical Genetics - Neuropsychiatric Genetics	105	354	362	T&A	Excluded	wrong outcome
**Sundram F., Murphy K.C.**	Velo-cardio-facial syndrome/22q11 deletion syndrome	2011	The SAGE Handbook of Developmental Disorders		219	238	T&A	Excluded	wrong publication type
**Shashi V., Berry M.N.**	Velocardiofacial syndrome (Chromosome 22q11.2 deletion syndrome) as a model of schizophrenia	2010	Secondary Schizophrenia		309	327	T&A	Excluded	wrong study design
**Ellez S., Braissand V., Knauer O.**	Velocardiofacial syndrome (deletion 22q11.2): Literature review and case study [Le syndrome vélo-cardio-facial (délétion 22q11.2): Une revue de littérature et présentation d'un cas clinique]	2004	Schweizer Archiv fur Neurologie und Psychiatrie	155	414	426	T&A	Excluded	wrong study design
**Usiskin S.I., Nicolson R., Krasnewich D.M., Yan W., Lenane M., Wudarsky M., Hamburger S.D., Rapoport J.L.**	Velocardiofacial syndrome in childhood-onset schizophrenia	1999	Journal of the American Academy of Child and Adolescent Psychiatry	38	1536	1543	T&A	Excluded	wrong outcome
**Márquez-Ávila C.S., Vizcaíno-Alarcón A., García-Delgado C., Núñez-Martínez P.M., Flores-Ramírez F., Reyes-de la Rosa A.D.P., Mendelsberg-Fishbein P., Ibarra-Grajeda D., Medina-Bravo P., Balderrábano-Saucedo N., Esteva-Solsona S., Márquez-Quiróz L.D.C., Flores-Cuevas A., Sánchez-Urbina R., Morales-Jiménez A.B., Garibay-Nieto N., Del Bosque-Garza J., Pietropaolo-Cienfuegos D., Gutiérrez-Camacho C., García-Morales L., Morán-Barroso V.F.**	Velocardiofacial syndrome in Mexican patients: Unusually high prevalence of congenital heart disease	2015	International Journal of Pediatric Otorhinolaryngolog y	79	1886	1891	T&A	Excluded	wrong outcome

**Van Geet C., Devriendt K., Eyskens B., Vermylen J., Hoylaerts M.F.**	Velocardiofacial syndrome patients with a heterozygous chromosome 22q11 deletion have giant platelets	1998	Pediatric Research	44	607	611	T&A	Excluded	wrong outcome
**Eliez S., Blasey C.M., Schmitt E.J., White C.D., Hu D., Reiss A.L.**	Velocardiofacial syndrome: Are structural changes in the temporal and mesial temporal regions related to schizophrenia?	2001	American Journal of Psychiatry	158	447	453	T&A	Excluded	wrong outcome
**Ruotolo R.A., Veitia N.A., Corbin A., McDonough J., Solot C.B., McDonald-McGinn D., Zackai E.H., Emanuel B.S., Cnaan A., LaRossa D., Arens R., Kirschner R.E.**	Velopharyngeal anatomy in 22q11.2 deletion syndrome: A three-dimensional cephalometric analysis	2006	Cleft Palate-Craniofacial Journal	43	446	456	T&A	Excluded	wrong outcome
**Havkin N., Tatum S.A., III, Shprintzen R.J.**	Velopharyngeal insufficiency and articulation impairment in velo-cardio- facial syndrome: The influence of adenoids on phonemic development	2000	International Journal of Pediatric Otorhinolaryngolog y	54	103	110	T&A	Excluded	wrong outcome
**Ysunza A., Carmen Pamplona M., Santiago Morales M.A.**	Velopharyngeal valving during speech, in patients with velocardiofacial syndrome and patients with non-syndromic palatal clefts after surgical and speech pathology management	2011	International Journal of Pediatric Otorhinolaryngolog y	75	1255	1259	FT	Excluded	incomplete outcome
**Simon T.J., Bearden C.E., McDonald Mc-Ginn D., Zackai E.**	Visuospatial and numerical cognitive deficits in children with chromosome 22q11.2 deletion syndrome	2005	Cortex	41	145	155	T&A	Excluded	wrong outcome
**Simon T.J., Ding L., Bish J.P., McDonald-McGinn D.M., Zackai E.H., Gee J.**	Volumetric, connective, and morphologic changes in the brains of children with chromosome 22q11.2 deletion syndrome: An integrative study	2005	NeuroImage	25	169	180	T&A	Excluded	wrong outcome

**Voormolen E.H.J., Wei C., Chow E.W.C., Bassett A.S., Mikulis D.J., Crawley A.P.**	Voxel-based morphometry and automated lobar volumetry: The trade-off between spatial scale and statistical correction	2010	NeuroImage	49	587	596	T&A	Excluded	wrong topic
**Owen M.J., Doherty J.L.**	What can we learn from the high rates of schizophrenia in people with 22q11.2 deletion syndrome?	2016	World Psychiatry	15	23	25	T&A	Excluded	wrong publication type
**Da Silva Alves F., Schmitz N., Bloemen O., van der Meer J., Meijer J., Boot E., Nederveen A., de Haan L., Linszen D., van Amelsvoort T.**	White matter abnormalities in adults with 22q11 deletion syndrome with and without schizophrenia	2011	Schizophrenia Research	132	75	83	T&A	Excluded	wrong outcome
**Villalon-Reina J., Jahanshad N., Beaton E., Toga A.W., Thompson P.M., Simon T.J.**	White matter microstructural abnormalities in girls with chromosome 22q11.2 deletion syndrome, Fragile X or Turner syndrome as evidenced by diffusion tensor imaging	2013	NeuroImage	81	441	454	T&A	Excluded	wrong outcome
**Sundram F., Campbell L.E., Azuma R., Daly E., Bloemen O.J.N., Barker G.J., Chitnis X., Jones D.K., van Amelsvoort T., Murphy K.C., Murphy D.G.M.**	White matter microstructure in 22q11 deletion syndrome: A pilot diffusion tensor imaging and voxel-based morphometry study of children and adolescents	2010	Journal of Neurodevelopmenta l Disorders	2	77	92	T&A	Excluded	wrong outcome
**Merico D., Zarrei M., Costain G., Ogura L., Alipanahi B., Gazzellone M.J., Butcher N.J., Thiruvahindrapuram B., Nalpathamkalam T., Chow E.W.C., Andrade D.M., Frey B.J., Marshall C.R., Scherer S.W., Bassett A.S.**	Whole-genome sequencing suggests schizophrenia risk mechanisms in humans with 22q11.2 deletion syndrome	2015	G3: Genes, Genomes, Genetics	5	2453	2461	T&A	Excluded	wrong outcome
**Tassabehji M.**	Williams-Beuren syndrome: A challenge for genotype-phenotype correlations	2003	Human Molecular Genetics	12	R229	R237	T&A	Excluded	wrong topic
**Kurahashi H, Tsuda E, Kohama R, Nakayama T, Masuno M, Imaizumi K, Kamiya T, Sano T, Okada S, Nishisho I**	Another Critical Region for Deletion of 22q11: A Study of 100 Patients	1997	American Journal of Medical Genetics	72	180	185	FT	Included	

**Carlson C, Papolos D, Pandita R. K, Faedda G. L, Veit S, Goldberg R, Shprintzen R, Kucherlapati R, and Morrow B.**	Molecular Analysis of Velo-Cardio-Facial Syndrome Patients with Psychiatric Disorders	1997	American Journal of Human Genetics	60	851	857	FT	Included	

*FT : Full text
*T&A: Title and abstract
