# Supplementary material for: Association between phenotype and deletion size in 22q11.2 microdeletion syndrome: systematic review and meta-analysis
Source: Orphanet J Rare Dis. 2019 Aug 9;14:195. doi: 10.1186/s13023-019-1170-x (PMC6688301; doi:10.1186/s13023-019-1170-x)
Supplement: Supplementary file 2 — Raw data from individual studies. Table with the frequency of phenotypic outcomes cross tabulated with information on deletion size and segment of deletion. (PDF 52 kb) [file 13023_2019_1170_MOESM2_ESM.pdf]

Study	Deletion size	CHD		PA	
		with	*without*	*with*	*without*
KURAHASHI (n=48)	***AB***	*4*	*1*	*5*	*0*
	AC	*0*	*0*	*0*	*0*
	AD	*41*	*2*	*10*	*33*
	Total	48		48	
CARLSON (n=23)	***AB***	*5*	*5*	*10*	*0*
	AC	*0*	*0*	*0*	*0*
	AD	*9*	*4*	*13*	*0*
	Total	23		23	
FERNANDEZ (CHD n=14 ; PA=12)	***AB*** / ***AC***	*4*	*3*	*6*	*0*
	AD	*3*	*4*	*6*	*0*
	Total	14		12	
MICHAELOVSKY (n=105)	***AB***	*1*	*3*	*3*	*1*
	AC	*3*	*1*	*3*	*1*
	AD	*67*	*30*	*80*	*17*
	Total	105		105	
WU (n=43)	***AB***	*0*	*3*	-	-
	AC	-	-	-	-
	AD	*10*	*30*	-	-
	Total	43		0	
MONTEIRO (n=39)	***AB***	*0*	*3*	*3*	*0*
	AC	-	-	-	-
	AD	*23*	*13*	*32*	*4*
	Total	39		39	
HWANG (n=77)	***AB***	*1*	*2*	-	-
	AC	-	-	-	-
	AD	*36*	*38*	-	-
	Total	77		0	
MLYNARSKI (n=949)	***AB***	*29*	*13*	-	-
	AC	*10*	*5*	-	-
	AD	*564*	*328*	-	-
	Total	949		0	
REPETTO (CHD n=216 ; PA n=217)	***AB***	*3*	*8*	*8*	*4*
	AC	*3*	*2*	*4*	*1*
	AD	*118*	*82*	*140*	*60*
	Total	216		217	
